# Peroxisome inspired hybrid enzyme nanogels for chemodynamic and photodynamic therapy

**DOI:** 10.1038/s41467-021-25561-z

**Published:** 2021-09-02

**Authors:** Xing Qin, Chu Wu, Dechao Niu, Limei Qin, Xia Wang, Qigang Wang, Yongsheng Li

**Affiliations:** 1 Lab of Low-Dimensional Materials Chemistry, Key Laboratory for Ultrafine Materials of Ministry of Education, Frontier Science Center of the Materials Biology and Dynamic Chemistry, School of Materials Science and Engineering, East China University of Science and Technology, Shanghai, P. R. China; 2 School of Chemical Science and Engineering, Tongji University, Shanghai, P. R. China

**Keywords:** Biomedical materials, Nanomedicine, Nanotechnology in cancer

## Abstract

Peroxisome, a special cytoplasmic organelle, possesses one or more kinds of oxidases for hydrogen peroxide (H_2_O_2_) production and catalase for H_2_O_2_ degradation, which serves as an intracellular H_2_O_2_ regulator to degrade toxic peroxides to water. Inspired by this biochemical pathway, we demonstrate the reactive oxygen species (ROS) induced tumor therapy by integrating lactate oxidase (LOx) and catalase (CAT) into Fe_3_O_4_ nanoparticle/indocyanine green (ICG) co-loaded hybrid nanogels (designated as FIGs-LC). Based on the O_2_ redistribution and H_2_O_2_ activation by cascading LOx and CAT catalytic metabolic regulation, hydroxyl radical (·OH) and singlet oxygen (^1^O_2_) production can be modulated for glutathione (GSH)-activated chemodynamic therapy (CDT) and NIR-triggered photodynamic therapy (PDT), by manipulating the ratio of LOx and CAT to catalyze endogenous lactate to produce H_2_O_2_ and further cascade decomposing H_2_O_2_ into O_2_. The regulation reactions of FIGs-LC significantly elevate the intracellular ROS level and cause fatal damage to cancer cells inducing the effective inhibition of tumor growth. Such enzyme complex loaded hybrid nanogel present potential for biomedical ROS regulation, especially for the tumors with different redox state, size, and subcutaneous depth.

## Abstract (web summary)

The control of reactive oxygen species in cancer cells is an attractive approach for anticancer applications. Here, the authors create a peroxisome inspired lactate oxidase and catalase loaded hydrogel with iron nanoparticles and NIR photosensitizer for glutathione activated chemodynamic and photodynamic therapy.

## Introduction

The study of pathological microenvironment based on the differences from normal tissues, such as different vascular abnormalities, oxidation state, pH value, and metabolic state, has become a prerequisite for the optimal treatment strategy. These specific changes play key roles in the exploring of biological mechanisms, drug screening, and the diagnosis and treatment of many diseases, especially tumors^1–3^. Among them, reactive oxygen species (ROS) are the active derivatives of oxygen metabolism in the microenvironment of all biological systems, which are also the first response of immune system to infection, stimuli, or injury, and are closely related to many diseases, including cancer, inflammatory response, atherosclerosis, asthma, and cystic fibrosis^4,5^.

As the successful explorations of ROS-related therapeutic strategy, the exogenous physical photodynamic therapy (PDT) and sonodynamic therapy (SDT) can produce cytotoxic singlet oxygen (^1^O_2_) for cancer therapy by the controllable light or ultrasound irradiation energy, transferred from the excited photosensitizers or sonosensitizers to oxygen molecules^6–8^. The endogenous pathological-responsive chemodynamic therapy (CDT) has begun focusing on utilizing inorganic nanoparticles (NPs) (e.g. Fe_3_O_4_, CuO, MnO) as nanoenzymes to stimulate the ROS and execute Fenton-like production of ·OH for tumor treatment without external instruments^9–14^. Comparatively, bioinspired by the enzyme bio-oxidation process in the marvelous neutrophils of innate immune systems, the enzyme dynamic therapy has been proposed in authors’ group, which can effectively convert the endogenous ROS (˙O_2_− and H_2_O_2_) into highly reactive ^1^O_2_ by the cascade biocatalytic reaction of loaded superoxide dismutase and chloroperoxidase responsively in the tumor region^15–17^.

Peroxisome is one special kind of diverse microbody, which is so named because it usually contains one or more enzymes, mainly oxidase, catalase, and peroxidase with H_2_O_2_ metabolism. Oxidase can oxidize substrates while reducing oxygen to H_2_O_2_. Besides, the marker enzyme as the “safety valve” of peroxisome is catalase, which can utilize the H_2_O_2_ to further oxidize a variety of toxic substrates by the peroxidative reaction, and simultaneously catalyzes excess H_2_O_2_ accumulates to H_2_O and O_2_. Interestingly, the peroxisome usually has different sets of enzymes in a single organism of various cell types and adapt remarkably to changing conditions^18,19^. Compared to the oxidation reactions of other oxidases, the in situ lactate oxidase (LOx) catalysis exhibits the higher efficiency due to the abundant lactate substrate in the hypoxia tumor microenvironment, as well as the LOx from facultative bacteria used in industrial production with the ultra-low O_2_ reaction threshold^20^. Recently, the studies based on the engineered LOx system provided the possibility of directly targeting the circulating milieu such as the lactate to alleviate intracellular redox imbalance between the inside and outside of cells^21,22^.

Bioinspired by the H_2_O_2_ activation reaction and the O_2_ redistribution pathways of peroxisome, herein as a proof of concept, we design and develop an enzyme complex-loaded hybrid nanogel to achieve ROS regulation by integrating LOx and catalase (CAT) into Fe_3_O_4_ NP loaded and indocyanine green (ICG) encapsulated hybrid nanogels (designated as FIGs-LC). By fully considering the ROS regulation of the LOx and CAT enzymes and their cascading catalytic metabolic regulation, the hydroxyl radical (·OH) and singlet oxygen (^1^O_2_) production can be modulated for glutathione (GSH)-activated CDT and NIR-triggered PDT of tumors. We envision that the hybrid nanogels can potentially fulfill the diverse functions for biomedical ROS regulation as a safe and efficient ROS-related tumor therapeutic strategy.

## Results

### Synthesis and characterization of the enzyme complex-loaded hybrid nanogels

**Fig. 1:**
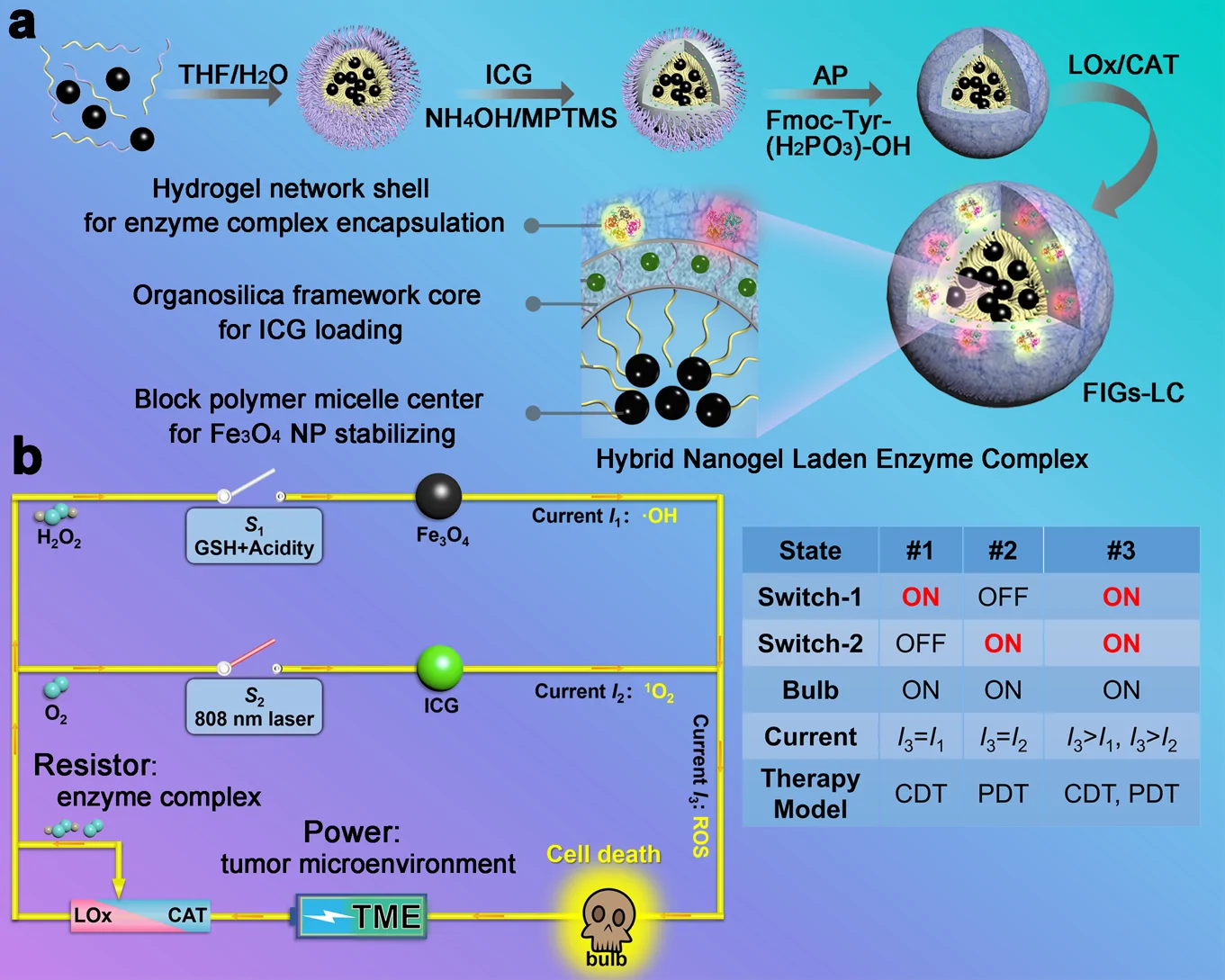
Schematics of synthesis and therapeutic mechanism of FIGs-LC. **a** The synthetic procedures of FIGs-LC include: (1) self-assembly of Fe_3_O_4_ NPs-encapsulated polystyrene-*block*-poly (acrylic acid) (PS-*b*-PAA) micelles (Fe_3_O_4_@PS-*b*-PAA) in selective solution; (2) the formation of indocyanine green (ICG)-loaded hybrid nanoparticles (Fe_3_O_4_@IHPs) by doping ICG and introducing organic silane 3-mercaptopropyltrimethoxysilane (MPTMS); (3) acid phosphatase (AP)-triggered hydrogel coating onto Fe_3_O_4_@IHPs, denoted with Fe_3_O_4_@IHPs@NanoGels (FIGs); (4) immobilization of lactate oxidase (LOx) and catalase (CAT) into FIGs, the final particles are denoted by FIGs-LC. **b** Schematic circuit diagram for the peroxisome-inspired therapeutic mechanism of FIGs-LC based on the dual-enzyme-regulated ROS generation with GSH and NIR activation: the intratumoral lactate and H_2_O_2_ are catalyzed by LOx and CAT (resistor regulation) to generate H_2_O_2_ and O_2_, respectively. Then, H_2_O_2_ is used to produce hydroxyl radicals (·OH, current *I*_1_) in the presence of Fe_3_O_4_ NPs within GSH-enriched acidic tumor microenvironment (Switch-1, *S*_1_), leading to the cancer cell death (bulb on). In the reaction, the produced O_2_ is converted into singlet oxygen (^1^O_2_, current *I*_2_) under the irradiation of an 808 nm laser (Switch-2, *S*_2_), which can also cause significant cell death (bulb on). Both CDT and PDT can be executed independently and induce the death of cancer cells, as well be activated simultaneously (*S*_1_ and *S*_2_ are connected) to achieve improved antitumor therapy.

The synthesis procedures for dual-enzyme-loaded hybrid nanogels are illustrated in Fig. 1a. Initially, hydrophobic Fe_3_O_4_ NPs are encapsulated into the hydrophobic cores of polystyrene-*block*-poly (acrylic acid) (PS-*b*-PAA) micelles through the hydrophobic interaction-induced self-assembly behavior, obtaining the as-prepared Fe_3_O_4_-loaded PS-*b*-PAA micelles (Fe_3_O_4_@PS-*b*-PAA). Afterwards, ICG and 3-mercaptopropyltrimethoxysilane (MPTMS) are simultaneously added into the above micellar solution to obtain Fe_3_O_4_/ICG co-loaded hybrid NPs (Fe_3_O_4_@IHPs). Under the alkaline catalysis (by adding NH_4_OH), MPTMS undergoes hydrolysis and condensation in the confined PAA shells of Fe_3_O_4_@PS-*b*-PAA micelle, generating the disulfide bonds (–S–S–)-connected and ICG-doped organosilica framework. To afford the efficient enzyme loading, a supramolecular hydrogel coating is introduced through the acid phosphatase (AP)-initiated dephosphorylation of *N*-(fluorenyl-methoxycarbonyl) tyrosine phosphate (Fmoc-Tyr(H_2_PO_3_)-OH), resulting in the formation of Fe_3_O_4_/ICG co-loaded hybrid nanogels (Fe_3_O_4_@IHPs@NanoGels, FIGs). Finally, the enzyme complex, LOx, and catalase are successively immobilized into the FIGs through charge adsorption effects to achieve the final construction of enzyme complex-loaded hybrid nanogels (Fe_3_O_4_@IHPs@NanoGels-LOx/CAT, FIGs-LC).

The FIGs-LC provide a paradigm to tune ROS production in antitumor therapy. As shown in Fig. 1b, this tunability is realized by adjusting the loading amounts of LOx and CAT (illustrated by a resistor), followed by the dynamic processes of CDT and PDT under different stimuli. In the acidic tumor microenvironment (TME), iron ions are gradually released from FIGs-LC by the trigger of endogenous reduced glutathione (GSH). Meanwhile, abundant lactate molecules are in situ catalyzed by LOx to increase localized H_2_O_2_ concentration, is sequentially devoted to generating highly cytotoxic ·OH with the aid of iron ions for realizing LOx-enhanced CDT. Based on the lactic acid metabolism regulation by LOx catalysis, the produced pyruvate can enter the cell to participate in the lactate dehydrogenase (LDH) reaction to facilitate further oxidation of NADH to NAD^+^^21,22^. In this cascade catalytic system, the utilization of endogenous H_2_O_2_ can alleviate oxygen deficiency by CAT biocatalytic conversion on one side, and the reduced oxygen consumption of tumor cells through LOx-CAT cascade biocatalytic conversion of lactate coupling with the pyruvate cycle can be achieved, because the decrease of NADH can counteract the O_2_ consumption in the later oxidative phosphorylation, as shown in Supplementary Fig. 1. Therefore, FIGs-LC with the LOx-CAT cascade system can catalyze the excess lactate substrate to H_2_O_2_ intermediate in the tumor hypoxia condition for GSH-activated CDT. By precisely manipulating the ratio of LOx and CAT, the H_2_O_2_ intermediate and endogenous H_2_O_2_ molecules are transferred to O_2_ molecules by CAT and form stable O_2_ nanobubbles in the peptide-assembled hydrogel networks. The enzymatic nanobubbles with high-density gas inside the nanodomains but the abnormal stability play the key role in effective coupled PDT. These produced O_2_ molecules immediately accept energy from the adjacent excited-state ICG molecules and are converted to ^1^O_2_ under an 808 nm laser irradiation; this spatiotemporally synchronous O_2_ supply is beneficial to promote ^1^O_2_ production of PDT. As an emerging treatment modality, the therapeutic efficacy of PDT is not only subject to the O_2_ concentration gradients within tumor tissues but also limited by the tissue penetration depth of laser. Excitingly, Fenton reaction is not constrained by penetration depth, produce lethal ·OH to kill the deep cancer cells, and be further enhanced by LOx. As a result, the production of ·OH and ^1^O_2_ can be readily modulated by simply varying the loading amounts of LOx or CAT. Finally, the dual-enzyme catalytic reactions result in conspicuous ROS elevation to induce the death of cancer cells, indicating the potential of FIGs-LC to tumor therapy by precise ROS regulation.

**Fig. 2:**
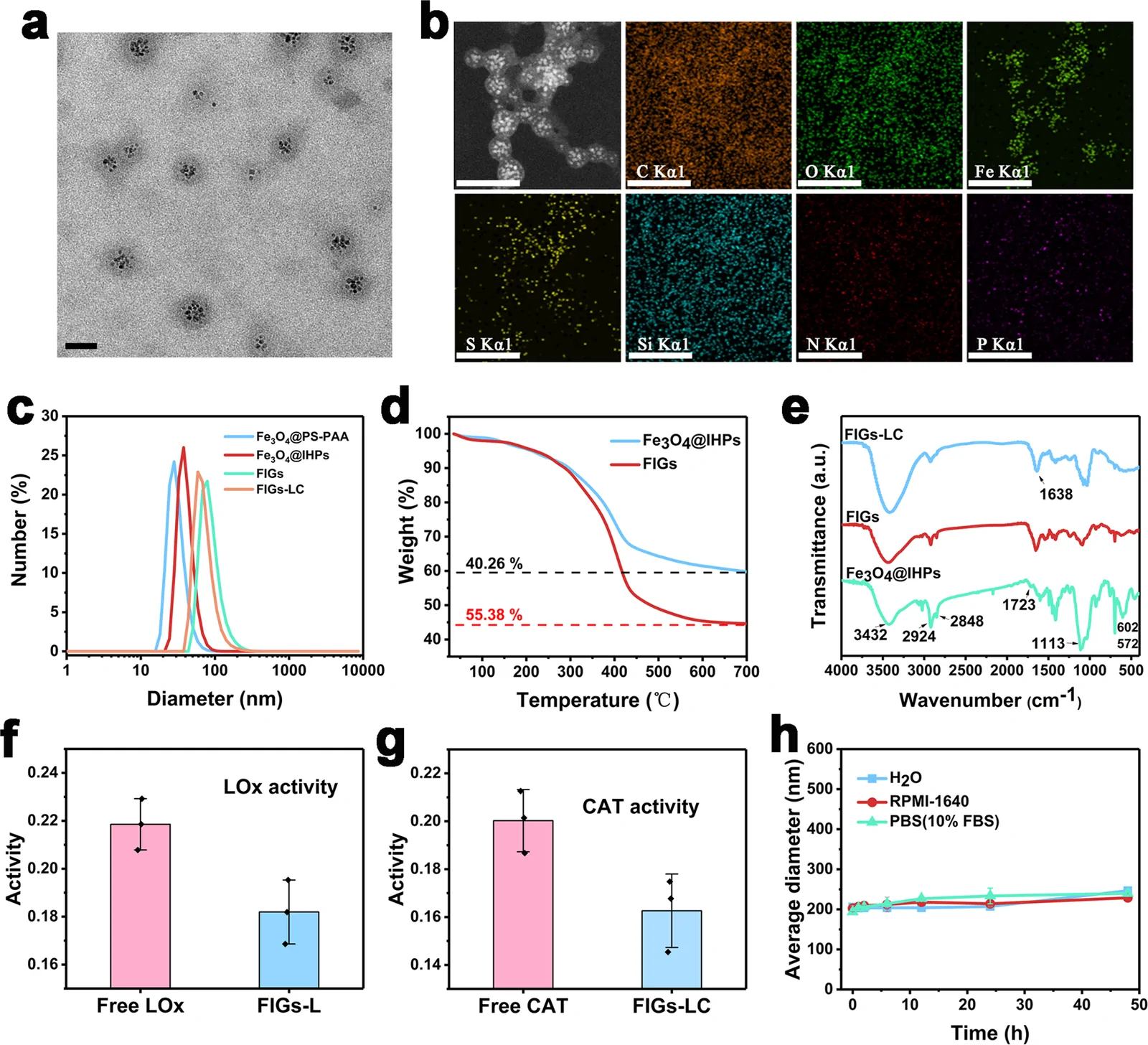
Characterizations of FIGs-LC. **a** TEM image of FIGs-LC, scale bar: 50 nm. **b** Elemental mappings of FIGs-LC, scale bar: 100 nm. **c** Hydrodynamic size distributions (Number) of Fe_3_O_4_@PS-*b*-PAA, Fe_3_O_4_@IHPs, FIGs, and FIGs- LC in water determined by DLS, respectively. **d** Thermogravimetric analysis (TG) curves of Fe_3_O_4_@IHPs, FIGs. **e** FT-IR spectra of Fe_3_O_4_@IHPs, FIGs, and FIGs-LC, respectively. **f** LOx activity of FIGs-L tested by a peroxidase-couple spectrophotometric assay, the absorbance (565 nm) of produced quinonediimine dye is positively related to H_2_O_2_ production (LOx activity). **g** CAT activity of FIGs-LC was reflected by the decreased absorbance of substrate (H_2_O_2_) at 240 nm. **h** Size stability (by Intensity) of FIGs-LC in water, culture medium (RPMI-1640), and PBS (pH = 7.4, contain 10% FBS) over time. In **f**–**h**, data are presented as mean ± s.d. from three independent replicates.

The structures of FIGs-LC were characterized by transmission electron microscope (TEM). As shown in Fig. 2a, the uniform Fe_3_O_4_ NPs within the hydrophobic core and a low-contrast hydrogel shell were clearly observed. The hydrogel shell on the surface of Fe_3_O_4_@IHPs was further confirmed by comparing the TEM and scanning electron microscopy images of Fe_3_O_4_@IHPs and FIGs (Supplementary Fig. 2). In comparison to the images of FIGs, the typical core–shell structures of the hybrid nanogels were well maintained after the immobilization of LOx and CAT. Element mapping analyses of FIGs-LC showed the uniform distributions of Fe element (from Fe_3_O_4_) and S element (from MPTMS) (Fig. 2b), indicating the successful encapsulation of Fe_3_O_4_ NPs and disulfide bonds-doped organosilica coating by MPTMS. Moreover, N and P element signals confirmed the distribution of dual-enzyme-loaded hydrogel shell on the Fe_3_O_4_@IHPs. To further prove the successful preparation of the hydrogel, a macro-hydrogel was synthesized by increasing the AP and Fmoc-Tyr (H_2_PO_3_)-OH amount (Supplementary Fig. 3).

In addition, the variations of hydrodynamic size and zeta potential in the preparation process for FIGs-LC were revealed. As presented in Fig. 2c, the average hydrodynamic sizes of FIGs and FIGs-LC are determined to be 88.42 and 72.91 nm, which means that the immobilization of enzymes did not deprave the good dispersity of FIGs. In addition, the size increase can be attributed to the slight expansion of hydrogel coating after the immobilization of enzymes. Moreover, to reflect the particle size of FIGs-LC more intuitively, the particle size distribution of FIGs-LC was further tested by the NanoSight technology analysis (NTA), which can capture the particle’s moving under Brownian motion. As shown in Supplementary Fig. 4, most of the particle sizes of FIGs-LC are smaller than 300 nm. The decreased zeta potential from −29.57 (Fe_3_O_4_@PS-*b*-PAA) to −33.33 mV (Fe_3_O_4_@IHPs) and the increase from −33.33 to −22.90 mV (FIGs-LC) further indicated the formation of organosilica shell and successful coating of hydrogel (Supplementary Fig. 5). Thermal analyses of Fe_3_O_4_@IHPs and FIGs showed that the weight proportion of the hydrogel was calculated to be 15.22% (Fig. 2d). Fourier transform infrared (FT-IR) spectra of Fe_3_O_4_@IHPs showed the distinguished absorption peak at 1113 cm^−1^ that was attributed to the Si–O–Si vibrations and the peak of 672 cm^−1^ derived from Fe–O vibrations (Fig. 2e)^23,24^. Noticeably, the stretching vibration of C = O bonds (1723 cm^−1^) assigned to the carboxyl groups of PAA blocks remained on the surface of Fe_3_O_4_@IHPs^25^, which is favorable for the formation of supramolecular hydrogel coating. Besides, the peaks at 3432, 2924, and 2848 cm^−1^ originated from the vibrations of –OH, –CH_2_, and –CH of organosilica. After the immobilization of LOx and CAT into FIGs, a characteristic peak of 1638 cm^−1^ derived from the enzymes was clearly observed, further verifying the successful loading of enzymes^15^.

To determine the loading capability and activity of the loaded dual enzyme, a peroxidase-couple spectrophotometric assay and UV-Vis spectrophotometer were employed. As presented in Supplementary Table 1, the loading efficiencies of LOx and CAT in FIGs-LC were determined to 96.88% and 84.83%, respectively. In addition, the corresponding loading amounts of LOx and CAT in FIGs-LC were 1.99 and 3.48 U/mg, respectively. Compared with free LOx, the activity of LOx in FIGs-L (without catalase) remained a very close level (Fig. 2f). Also, the catalytic H_2_O_2_ decomposition by FIGs-LC remained at an appreciable level compared with free CAT in 60 s (Fig. 2g). Since the in vivo TME is slightly acidic, it is necessary to evaluate the enzymatic activities and stability of FIGs-LC at different pH values (7.0 and 6.0). As depicted in Supplementary Fig. 6a, b, the activity of LOx showed an activity of 73% at pH 6.0, which was comparable to that at pH 7.0. In addition, the activity of loaded CAT remained 89% at pH 6.0 in comparison to the activity at pH 7.0. At room temperature, the relative activities of both LOx and CAT decreased quite slowly with time, suggesting the applicable storage stability (Supplementary Fig. 6c, d). As identified by dynamic light scattering (DLS) (Fig. 2h), no obvious change on the average hydrodynamic size of FIGs-LC in water, culture medium (RPMI-1640), and PBS (contain 10% fetal bovine serum, FBS) were found over 2 days, showing its excellent dispersity and stability. Furthermore, no significant enzyme release was observed over even 28 days (Supplementary Fig. 7). Besides, compared to free ICG, the stability of ICG in FIGs-LC was greatly improved (Supplementary Fig. 8) owing to the protection effect from the organosilica framework. More importantly, compared with free ICG, the maximum UV absorption wavelength of FIGs-LC shifted to 808 nm (Supplementary Fig. 9), demonstrating that FIGs-LC can serve as higher ^1^O_2_-yielding photosensitizers with the full use of commercial 808 nm laser.

### Responsive behaviors to TME

**Fig. 3:**
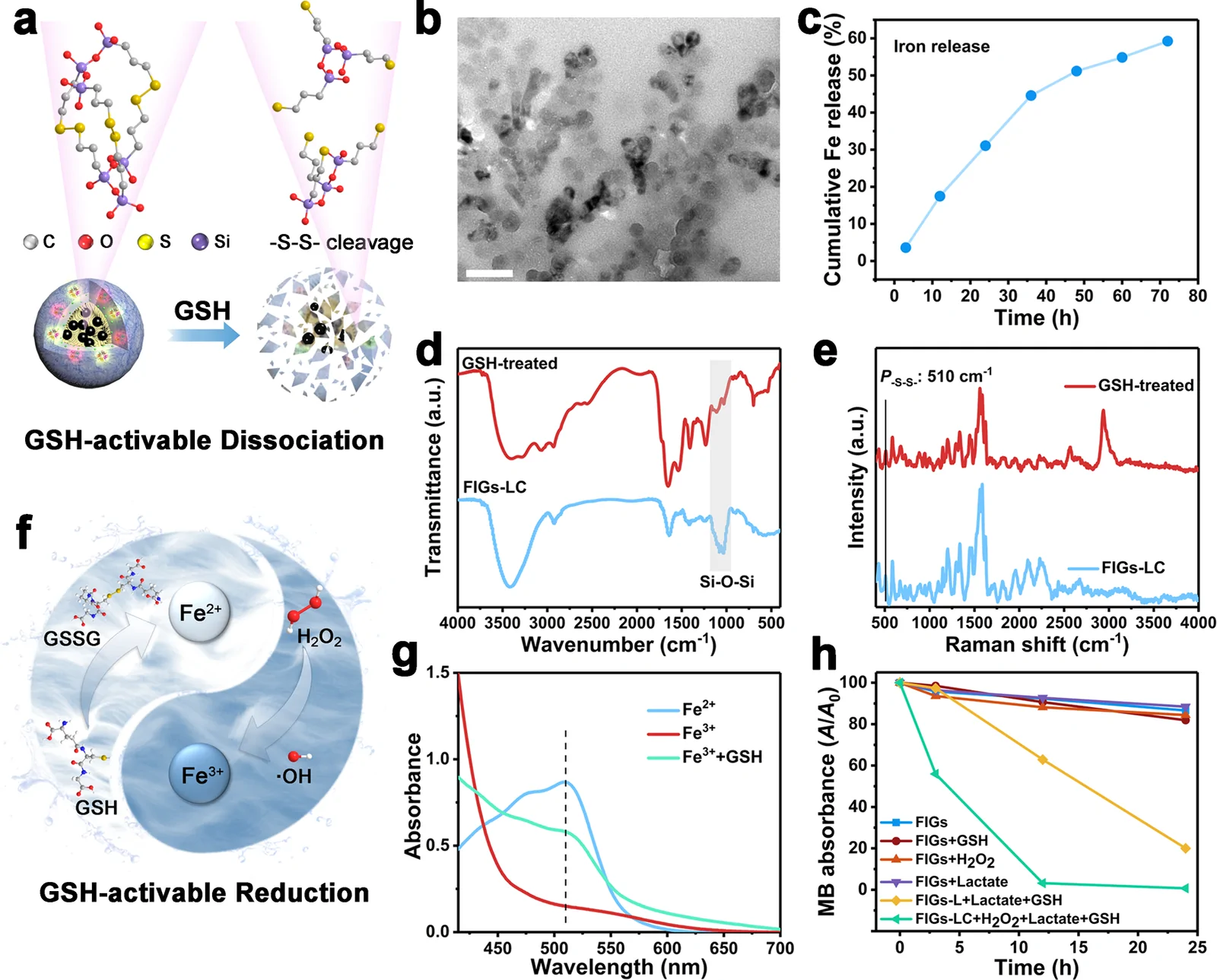
Responsive behaviors of FIGs-LC to tumor microenvironment. **a** Schematic of GSH-induced –S–S– cleavage, leading to the collapse of FIGs-LC and iron release. **b** TEM images of FIGs-LC after a 5-day incubation in the simulated TME solutions (containing 100 μM of H_2_O_2_, 10 mM of GSH, and 2 mM of lactate, pH 6.0), scale bar:100 nm. **c** Cumulative Fe ions release from FIGs-LC in the simulated TME solutions. **d** FT-IR spectra of the FIGs-LC and GSH-treated FIGs-LC. **e** The Raman shift of the FIGs-LC and GSH-treated FIGs-LC. **f** Schematic cycle of Fe^3^^+^ and Fe^2^^+^ in the tumor microenvironment. **g** UV-Vis spectra of Fe^2^^+^ solution and Fe^3^^+^ solution after adding 1,10-phenanthroline monohydrate. Specially, the emerging peak at 510 nm indicated the Fe^3^^+^ was converted into Fe^2^^+^ by GSH. **h** The MB degradation (*A*/*A*_0_) curves under different conditions.

The disulfide bonds (–S–S–) in the organosilica shell not only act as covalent linkers of Si–O–Si frameworks, but also serve as intelligent gates with GSH-responsive property. In the present work, the disulfide bonds-doped organosilica shell makes the FIGs-LC intelligent nanomedicines with GSH-activable CDT based on the over-expression of GSH in TME. As illustrated in Fig. 3a, the GSH in TME can break the disulfide bonds in organosilica framework, further leading to the collapse of FIGs-LC and sequential iron release. The particle collapse and consequent aggregation triggered by GSH in early incubation were evidenced by TEM (Fig. 3b and Supplementary Fig. 10) and the hydrodynamic diameter changes in PBS with varied GSH concentrations (0, 1, 5, and 10 mM, pH 6.0) (Supplementary Fig. 11) were recorded. However, with the duration period further prolonged to 10 days, Fe_3_O_4_ NPs and large-sized aggregations gradually disappeared. This whole degradation progress was accompanied by the release of iron ions, and the release amount (including Fe^2^^+^ and Fe^3^^+^) under normal physiological milieu and TME was measured by a spectrophotometric method using 1,10-phenanthroline monohydrate. The formed stable complex [Fe(phen)_3_]^2^^+^ of Fe^2^^+^ and 1,10-phenanthroline presented a characteristic absorption peak at the wavelength of 510 nm in UV-Vis spectra, and its standard curve was obtained by the absorbance of Fe^2^^+^ solutions at different concentrations (Supplementary Fig. 12)^26^. After incubated in the simulative TME (containing 100 μM of H_2_O_2_, 10 mM of GSH, and 2 mM of lactate, pH 6.0), iron ions were gradually released from FIGs-LC and the cumulative release amount was determined to be 59.25% for 72 h (Fig. 3c). In contrast, none of Fe^2^^+^ was detected under normal conditions by a UV-Vis spectrophotometer (Supplementary Fig. 13), which corresponds to the high stability of FIGs-LC in PBS. Differing from other iron-transport strategies such as using hydrophilic Fe_3_O_4_ NPs or the coordination of organic groups and iron ions, the organosilica protected hydrophobic Fe_3_O_4_ NPs in the present system are very hard to release iron ions under normal conditions. The collapse of organosilica framework was verified by FT-IR spectra, which showed that the absorption peak of Si–O–Si vibrations at about 1110 cm^−1^ declined after GSH incubation (Fig. 3d). In the meantime, the Raman shift of disulfide bonds (510 cm^−1^) was detected in FIGs-LC and obviously weakened after the incubation with 10 mM of GSH due to the breakage of –S–S– bonds (Fig. 3e)^27^.

It is generally reported that the Fenton reaction rate between Fe^2^^+^ and H_2_O_2_ is significantly higher than that of Fe^3^^+^ ions^28^. However, Fe^2^^+^ is easily oxidized into Fe^3^^+^ in the preparation and delivery processes. Thus, we designed a Fe^2^^+^/Fe^3+^ cycle, as depicted in Fig. 3f, where the low active Fe^3^^+^ are reduced to Fe^2^^+^ by reductive GSH and the GSH is oxidized into GSSG^29^, highly active Fe^2^^+^ is conducive to ·OH formation in the tumor tissue. To prove this assumption, a control experiment involving the Fe^3^^+^-to-Fe^2^^+^ conversion by 1,10-phenanthroline was conducted (Fig. 3g). With the addition of 1,10-phenanthroline into the Fe^3^^+^ solution, the characteristic peak of [Fe(phen)]^2^^+^ complex was not detected; however, a peak at 510 nm was observed when it was added into Fe^2^^+^ solution, indicating the sensitive and specific detection for Fe^2^^+^. However, once GSH was introduced into the Fe^3^^+^ solution, the characteristic peak at 510 nm immediately emerged (Fig. 3g), suggesting the occurrence of Fe^3^^+^/Fe^2^^+^ conversion triggered by GSH. To confirm the trigger effect of GSH for CDT, the GSH-responsive ·OH generation capacity of as-prepared nanogels were investigated by monitoring the degradation rates of methylene blue (MB). As shown in Fig. 3h, it is found that Fenton reaction took place in the solutions with co-existence of FIGs-LC (Fe_3_O_4_ concentration is 15 μg/mL) and H_2_O_2_ (100 μM) and GSH (10 mM). Besides, ·OH was observed in FIGs-L (without CAT) after replacing H_2_O_2_ by lactate due to the lactate-to-H_2_O_2_ conversion, demonstrating the efficient catalytic performance of loaded LOx. It’s worth noting that the MB absorbance declined slightly only in the normal physiological environment (Supplementary Fig. 14), which is associated with the instability of MB, revealing that the Fenton reaction is in “OFF” state under normal conditions.

### ROS regulation by the enzyme complex-loaded hybrid nanogel system

**Fig. 4:**
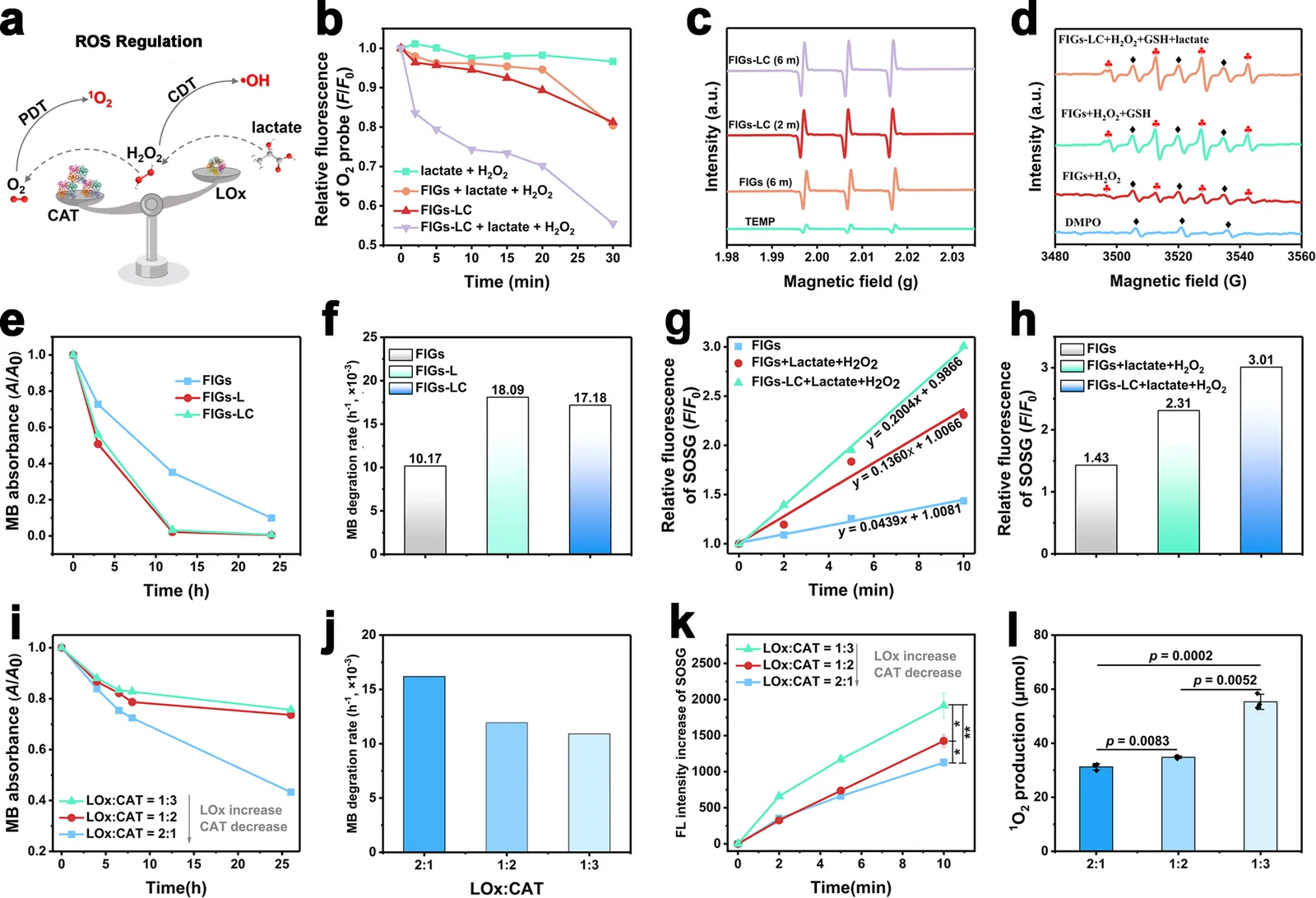
ROS regulation by FIGs-LC. **a** Schematic ROS regulation of LOx-modulated ·OH and CAT-modulated ^1^O_2_ production. **b** Changes (*F*/*F*_0_) in the fluorescence intensity of O_2_ probe (Ru(dpp)) over time. **c** ESR spectra of ^1^O_2_ with TEMP as trapper. **d** ESR spectra of ·OH with DMPO as trapper, red clubs: hydroxyl free radicals; black diamonds: oxidized DMPO radicals. **e** Degradation (*A*/*A*_0_) of methylene blue (MB) over FIGs, FIGs-L, and FIGs-LC in the simulated TME solutions with pH 6.0 (100 μM of H_2_O_2_, 10 mM of GSH, and 2 mM of lactate). **f** Relative degradation rates of MB according to the iron concentration. **g** Relative fluorescence intensity of SOSG as a function of laser irradiation time for the FIGs and FIGs-LC. **h** Fluorescence intensity of SOSG at 10 min for the FIGs and FIGs-LC. **i** The MB degradation curve of FIGs-LC with the different loading amount of LOx and CAT (1:3, 1:2, 2:1, LOx:CAT, U/U) under the simulated tumor microenvironment. **j** MB degradation rates of FIGs-LC with different loading ratios between LOx and CAT, and each histogram represents the mean ± s.d. **k** The fluorescence changes of ^1^O_2_ probe (SOSG) of FIGs-LC with different loading amounts of LOx and CAT (1:3, 1:2, 2:1, LOx:CAT, U/U) under the simulated tumor microenvironment. **l** The ^1^O_2_ production of FIGs-LC with different loading ratios between LOx and CAT at 5 min. In **k** and **l**, data are presented as mean ± s.d. from three independent replicates; *p* values were analyzed by Student’s two-sided *t*-test.

The integration of LOx and CAT endows FIGs-LC with peroxisome-like functions: LOx selectively oxidizes the endogenous lactate and produces H_2_O_2_, then the produced H_2_O_2_ and endogenous H_2_O_2_ are degraded to O_2_ by CAT. As illustrated in Fig. 4a, the CDT and PDT outcomes can be regulated by controlling the loading amounts of LOx or CAT. In details, with the increasing of CAT, more H_2_O_2_ molecules will be catalyzed into O_2_, and thus more ^1^O_2_ are produced, the ·OH production by CDT is correspondingly reduced. On the contrary, an increasing LOx amount will result in more H_2_O_2_ production and consume more O_2_, thus promote ·OH generation and oppositely decrease ^1^O_2_ production.

The production of O_2_ in buffer solution containing FIGs-LC, as a result of H_2_O_2_ decomposition catalyzed by LOx, was monitored by a fluorescence probe. The fluorescence of tris (4,7-diphenyl-1, 10-phenanthroline) ruthenium (II) dichloride (Ru (dpp)) can be quenched immediately by the newly produced O_2_ molecules. As shown in Fig. 4b, the dramatic fluorescence decreased of Ru (dpp) in group of FIGs-LC+ lactate + H_2_O_2_ confirmed that FIGs-LC produced considerable O_2_ molecules and led to the quenching of neighboring Ru (dpp) molecules, confirming the O_2_ generating capability of FIGs-LC. By comparison, there were very slight fluorescence declines were observed in group of FIGs-LC (without lactate and H_2_O_2_) and group of FIGs + lactate + H_2_O_2_ (without LOx and CAT) due to the concentration fluctuation of pre-existing oxygen. In this cascade catalytic system, the endogenous H_2_O_2_ and H_2_O_2_ intermediate can be efficiently transferred to O_2_ molecules to form the stable O_2_ nanobubbles in the hydrophobic microdomain of peptide-assembled supramolecular hydrogel by cascade CAT. It is reported that the nanobubbles exhibit the special characterization, with high-density gas but showing abnormal stability^30–32^. To address this issue, the efficient generation of O_2_ nanobubbles was tested by the NanoSight technology and synchrotron-based scanning transmission X-ray microscopy (STXM) analysis. As shown in Supplementary Fig. 15, 100 µM H_2_O_2_ as the control was heated and decomposed to produce O_2_, which displayed the lower particle concentration since the relatively low and diffused O_2_ molecules cannot reach the saturation concentration to form the detectable bubbles. STXM results (Supplementary Fig. 16) further verified the highly condensed oxygen gas molecules trapped as hydrogel-stabilized nanobubbles, with the high oxygen density inside but also the excellent stability (4 to 6 hours in an open solution system). These hydrogel-stabilized enzymatic O_2_ nanobubbles, maintain a dense state instead of the ideal gas diffused state, would contribute to the efficient PDT under hypoxic conditions. Given the photothermal effect of ICG may interfere with the detection of ^1^O_2_ in photodynamic process, the photothermal performances of FIGs-LC under different laser powers were recorded. As shown in Supplementary Fig. 17, upon a 0.25 W laser irradiation, all the FIGs-LC solutions with different ICG concentrations (4, 8, and 16 μg/mL) underwent slight temperature rises (<6 °C) within 5 min; hence, 0.25 W is the desired laser power for follow-up ^1^O_2_ observations.

The qualitative detection of ^1^O_2_ was accomplished by the electron spin resonance (ESR) method with 2,2,6,6-tetramethylpiperidine (TEMP) as a trapping agent. As shown in Fig. 4c, after exposure to the 808 nm laser for 6 min, the typical 1:1:1 peaks of ^1^O_2_ were observed in the FIGs spectra and the intensity was further intensified after loading the enzyme complex (FIGs-LC). For Fenton reaction, the ESR spectroscopy with 5,5-dimethyl-1-pyrroline-*N*-oxide (DMPO) as trapping agent was utilized to detect ·OH. As demonstrated by the characteristic 1:2:2:1 hydroxyl radical peaks, the enzyme-loaded FIGs-LC were more inclined to generate ·OH compared with FIGs in the buffer saline simultaneously containing lactate, H_2_O_2_, and GSH (Fig. 4d). The red clubs indicated the signals of hydroxyl free radicals, and some black diamonds labeled signals were also detected due to the presence of oxidized DMPO radicals^33^. In addition, more ·OH of FIGs were harvested in the GSH-contained FIGs solutions compared with that in GSH-free FIGs solutions, indicating that the participation of GSH was firmly pivotal in initiating Fenton reaction and improving the therapeutic efficacy of CDT. In the same incubation condition (100 μM of H_2_O_2_, 10 mM of GSH, and 2 mM of lactate, pH 6.0), both FIGs-L and FIGs-LC exhibited stronger MB bleaching ability compared with FIGs due to the absence of LOx (Fig. 4e). More accurately, based on the Eq. (1) that defined in the “Methods” section, the relative MB degradation rates (MB relative to Fe) were calculated according to the MB concentration in solution (Supplementary Fig. 18) and iron concentration determined by inductively coupled plasma optical emission spectrometer. In contrast to FIGs, both FIGs-L and FIGs-LC presented faster degradation rate (Fig. 4f), indicating the relatively high ·OH generation due to the continuous H_2_O_2_ supply by LOx.

To better understand the enzyme-enhanced PDT mechanism, singlet oxygen sensor green (SOSG) was used in the quantification for ^1^O_2_. As demonstrated in Fig. 4g, the slopes of time-dependent SOSG fluorescence curves represented the generation rates of ^1^O_2_ under different conditions, and a higher slope means a faster generation rate. Apparently, in the presence of lactate (2 mM) and H_2_O_2_ (100 μM), FIGs-LC produced more ^1^O_2_ than FIGs as the highest fluorescence increasement reflected in Fig. 4h, indicating the enzyme activity of FIGs-LC remains stable and effective even in the harsh TME.

As the intelligent nanomedicine, it is essential to comprehensively study the ROS regulation ability of FIGs-LC and the regulation mechanism. For this purpose, a series of FIGs-LC with different enzymatic feeding ratios (1:3, 1:2, 2:1, LOx: CAT, U/U) were synthesized. Accordingly, MB degradation experiments and SOSG-based ^1^O_2_ detection for different FIGs-LC were recorded so that the ·OH and ^1^O_2_ production can be compared directly. As expected, increasing the loading amount of LOx (the loading amount of CAT decreased relatively) led to the raises in ·OH production, which was reflected by the obvious slumps of MB degradation rate and could be explained by the increased H_2_O_2_ formation and reduced H_2_O_2_ decomposition (Fig. 4i, j). Correspondingly, the consumption of dissolved O_2_ for lactate oxidation was worsened and thus causes the slowdown of ^1^O_2_ production. SOSG was applied to evaluate the ^1^O_2_ production of FIGs-LC under laser irradiation, as expected, the ^1^O_2_ generation rate of FIGs-LC increased in the order of 1:3 > 1:2 > 2:1 (LOx:CAT), according to the SOSG fluorescence curves (Fig. 4k). The FIGs-LC with the lowest loading of LOx (LOx: CAT = 1:3) presented the highest ^1^O_2_ generation rate due to the lowest O_2_ consumption and highest O_2_ generation and oppositely the FIGs-LC with the highest loading of LOx (LOx: CAT = 2:1) presented the lowest ^1^O_2_ generation rate at 5 min (Fig. 4l). Based on the above results, it can be concluded that the ·OH and ^1^O_2_ production of FIGs-LC can be easily adjusted by varying the loading amount of LOx or CAT.

### Intracellular characterization for synergistic CDT and PDT

Although FIGs produce a small quantity of ^1^O_2_ and ·OH with the dual stimuli of exogenous NIR laser and endogenic TME, the ROS level is too low to kill tumor cells drastically. To amplify the ^1^O_2_ and ·OH generation, both LOx and CAT enzymes were introduced for the increase of ROS level. To investigate the cellular uptake of the hybrid nanogels, flow cytometry (FC) and confocal laser scanning microscopy (CLSM) were employed. However, considering the maximum emission wavelength of ICG in FIGs-LC exceeds 800 nm, which is inconvenient for FC and CLSM analysis. An equal amount of Rhodamine B (RhB) instead of ICG was doped into the organosilica frameworks (the obtained hybrid nanogels are abbreviated as FRGs-LC) by the same procedures, with the emission wavelength at ≈585 nm (Supplementary Fig. 19). FC results showed a continuous intensity improvement of intracellular FRGs-LC fluorescence with prolonging incubation period (Supplementary Fig. 20). Visual observations through CLSM on human hepatocarcinoma cells (SMMC-7721) that incubated with FIGs-LC also showed a distinct red fluorescence derived from RhB (Supplementary Fig. 21). These results confirmed the effective internalization of FRGs-LC by cancer cells as precondition for sequential reactions.

**Fig. 5:**
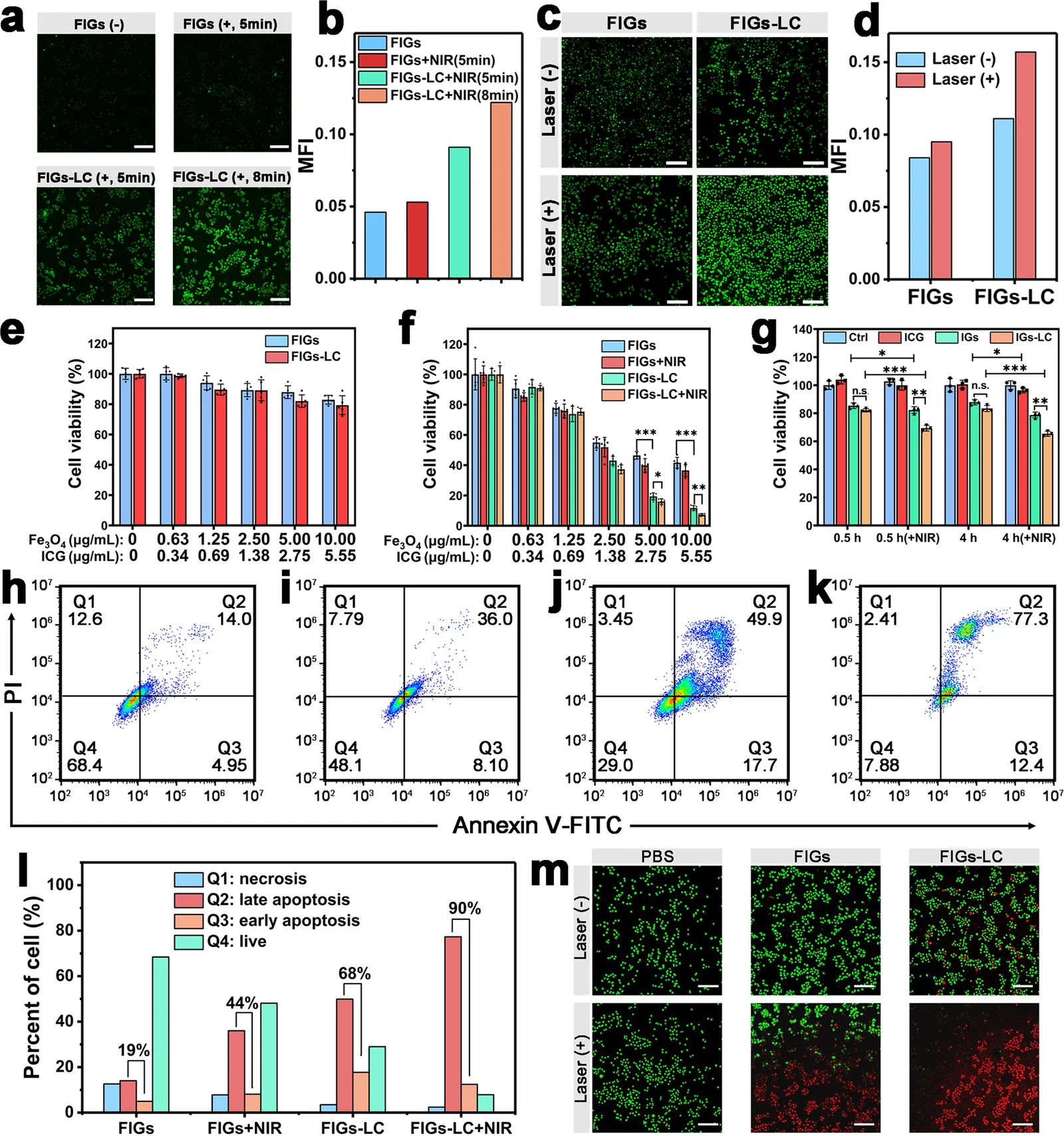
Intracellular characterization for synergistic CDT and PDT. **a** Confocal laser scanning microscopy (CLSM) images and corresponding mean fluorescence intensity (**b**) of human hepatocarcinoma cells (SMMC-7721) cells after co-incubation with FIGs or FIGs-LC for 6 h and treated with SOSG probe, “−” represents without laser irradiation and “+” represents with laser irradiation, scale bar: 200 μm. **c** CLSM images and corresponding mean fluorescence intensity (**d**) of SMMC-7721 cells treated with FIGs, and FIGs-LC for 12 h with DCFH-DA as a ROS detector, scale bar: 200 μm. **e** Cell viabilities of NIH-3T3 cells after incubation with FIGs or FIGs-LC for 48 h. **f** Cell viabilities of SMMC-7721 cells after incubation with FIGs or FIGs-LC for 48 h with or without NIR irradiation (NIR: 808 nm, 0.25 W, 5 min). **g** Cell viabilities of SMMC-7721 cells under hypoxic condition after incubation with ICG, IGs, or IGs- LC, the NIR irradiation was carried out after 0.5 or 4 h co-incubation (NIR: 808 nm, 0.25 W). In **e**–**g**, data are presented as mean ± s.d. from five (**e**, **f**) or three (**g**) independent replicates, *p* values were analyzed by Student’s two-sided *t*-test (*p < 0.05, **p < 0.01, ***p < 0.001, n.s. represents no significant differences). **h**–**k** Cell apoptosis of SMMC-7721 cells with FIGs, FIGs + NIR, FIGs-LC, and FIGs-LC + NIR incubation was determined by Annexin V-FITC/PI staining. **l** Quantitation of the cell apoptosis of SMMC-7721 cells under different treatments; the percent represents Q2 + Q3. **m** CLSM images of live/dead staining of SMMC-7721 cells after incubation with PBS, FIGs, FIGs-LC for 12 h with or without laser irradiation, live cells were stained with Calcein-AM and dead cells were stained with PI, scale bar: 200 μm.

The enzyme complexes can directly target the circulating milieu such as the lactic acid that rapidly exchange between the inside and outside of cells to disturb the intracellular redox imbalance. The lactate/pyruvate metabolism can directly influence the NADH/NAD^+^ pathway, and the mitochondrial overload of NADH determines the production of ROS for mitochondrial dysfunction. As proof of concept, the NADH/NAD^+^ redox ratio of SMMC-7721 cells has been explored after co-incubation with different groups (Supplementary Fig. 22). The results show that both the FIGs-L and FIGs-LC can increase the ratio of NADH/NAD^+^ redox, which can eventually elevate cellular levels of ROS to induce both higher early apoptosis and late apoptosis percent, indicating the imbalance of the redox ratio and the disturbing of ROS threshold level to be more vulnerable to further oxidative stress induced by ROS stimuli (Supplementary Fig. 23). Furthermore, the intracellular O_2_ level was investigated by co-incubating the Ru(dpp)-stained SMMC-7721 cells with different solutions (PBS, FIGs, FIGs-L, FIGs-LC) at different time points in the simulated hypoxic environment in a closed anoxic bag (1% O_2_ + 99% CO_2_). As shown in the Supplementary Fig. 24, a remarkable decrease in fluorescence signals of the FIGs-LC group after incubation was found, verifying the obvious increase of intracellular O_2_ level, especially in the first 0.5 and 1 h in this closed anoxic condition. This can be attributed to the CAT biocatalytic conversion of limited endogenous H_2_O_2_ and H_2_O_2_ intermediate without continuous atmosphere renewal. Meanwhile, the rapidly generated and hydrogel-stabilized enzymatic O_2_ nanobubbles can be detected and utilized more effectively. The cellular ^1^O_2_ and ROS levels on SMMC-7721 cells after incubation with FIGs-LC were further observed by CLSM. Once the FIGs-LC endocytosed by cancer cells, abundant stable O_2_ nanobubbles were primarily produced by CAT and H_2_O_2_ level was increased under the catalysis oxidation by LOx based on the lactate (2.0 mM)/H_2_O_2_(100 μM)-containing mimic culture medium, thereby promoting the ·OH and ^1^O_2_ generation and eventually induce the cell death. The cellular ^1^O_2_ was visualized by SOSG, and green fluorescence of SOSG was observed in cancer cells with FIGs incubation after irradiation with an 808 nm light irradiation (Fig. 5a, b). More interestingly, FIGs-LC exhibited a stronger green fluorescence under identical conditions and it further increased with the duration of the laser irradiation, implying the enzyme-enhanced ^1^O_2_ production. To evaluate the ultimate therapeutic potentials of FIGs-LC, 2′,7′-dichlorofluorescin diacetate (DCFH-DA) was used to monitor the ROS level in cancer cells (Fig. 5c, d and Supplementary Fig. 25). In the absence of laser irradiation (represented by laser (−)), ·OH was the only component of ROS, which is consistent with the previous MB degradation experiments and ESR results. As a result, FIGs-LC showed higher ROS fluorescence intensity than that of FIGs because of the LOx-enhanced Fenton reaction. In contrast, under the irradiation of an 808 nm laser at the same incubation conditions (represented by Laser (+)), the ROS fluorescence intensity of tumor cells further increased evidently due to the presence of additional ^1^O_2_ generation. All these results together suggest that a considerable amount of ROS is generated by FIGs-LC and can be further promoted by the enzyme complexes, which is expected to release its potential capability to kill cancer cells.

The cytotoxicities of FIGs and FIGs-LC to cancer SMMC-7721 cells, as well as to normal cells of mouse fibroblast cell line (NIH-3T3), were then examined by a cell counting method. As shown in Fig. 5e, no obvious proliferation inhibitions on NIH-3T3 cells were found within 48 h, indicating that the FIGs-LC are innocuous nanomedicines for normal cells in cancer therapy. On the contrary, due to the remarkable disparity that includes GSH and pH between normal tissues and tumor tissue, the FIGs-LC are expected to kill cancer cells in large amounts due to the TME-responsive CDT and the implementation of PDT. After the incubation with FIGs, the cell viabilities of SMMC-7721 cells obviously declined (42% viability at maximal concentration) and exhibited a concentration-dependent behavior (Fig. 5f), from which we can speculate that a tremendous amount of ·OH was generated in cancer cells by the Fenton reaction. As expected, consistent with the proposed process, the dual-enzyme-loaded FIGs-LC exhibited a greater proliferation suppression on cancer cells due to the amplified ·OH production (12% viability at maximal concentration). When exposed to an 808 nm laser, the cell viabilities were further reduced to 7.23% (FIGs-LC + NIR) owing to the CAT-triggered O_2_ production and the photo-induced massive ^1^O_2_ production. Generally, the toxicity of H_2_O_2_ in the normal cells and the dependence on substrate concentration restrict the performance of the enzyme-related treatment. As a control experiment, LOx alone (FIGs-L) without CAT killed both the normal and tumor cells because of the produced H_2_O_2_ (Supplementary Fig. 26). This reveals that CAT can play the key role in the effective detoxification during the metabolism of H_2_O_2_. Besides, the O_2_ synergistic PDT was further conducted by preparing hybrid nanogels without Fe_3_O_4_ NPs (IGs) and the enzyme complex-loaded hybrid nanogels without Fe_3_O_4_ NPs (IGs-LC) as the control groups. The cell viabilities of SMMC-7721 cells after incubation with ICG, IGs or IGs-LC were tested and results are shown in Fig. 5g. Upon laser irradiation, IGs-LC showed a better cell-killing capability compared to IGs due to the higher ^1^O_2_ production, indicating the significant intracellular O_2_ generation in hypoxic conditions. Exogenous PDT or endogenous CDT patterns can be therefore regulated according to different tumor types based on the ROS regulation.

Furthermore, the in vitro cell apoptosis assays of cancer cells under different incubation conditions (FIGs, FIGs + NIR, FIGs-LC, and FIGs-LC + NIR, respectively) were analyzed by FC. As shown in Supplementary Fig. 27 and Fig. 5h–k, cell populations were divided into four quadrants: live cells (Q4), early apoptotic cells (Q3), late apoptotic cells (Q2), and necrotic cells (Q1). In the groups of FIGs-LC (Fig. 5j) and FIGs-LC + NIR (Fig. 5k), majority of the cells was distributed in Q3 and Q2, demonstrating that the synergistic CDT and PDT caused significant apoptosis of cancer cells. The apoptotic rates (Q3 + Q2) of four groups were quantitatively analyzed and compared. As depicted in Fig. 5l, the cells in the group of FIGs-LC exhibited a higher apoptosis rate (68%) compared to those in the group of FIGs (19%), which confirmed again the enzyme-enhanced Fenton reaction due to the locally accelerated H_2_O_2_ generation. More interestingly, the cellular apoptosis rate in the group of FIGs increased from 19 to 44% with laser irradiation, while a greater increase from 68 to 90% was observed in the group of FIGs-LC, suggesting the light-activated PDT indeed induced cell apoptosis and was enhanced by the loaded enzyme complex in TME. In addition, the cytotoxicity was visually presented by a live/dead cell staining method (Fig. 5m). In details, SMMC-7721 cells were incubated with PBS (control), FIGs, and FIGs-LC for 12 h, followed by the laser irradiation for 5 min or not, and then the cells were stained with Calcein-AM (live cells, green fluorescence) and propidium iodide (dead cells, red fluorescence). In the absence of laser irradiation, a small number of red dead cells appeared in the group of FIGs-LC, compared to that in group of FIGs, which implied the enzyme-promoted Fenton reaction. And this low mortality was ascribed to the insufficient H_2_O_2_ accumulation in just 12 h. Comparatively, much more dead cells were intuitively discerned when treated with FIGs-LC plus laser irradiation (FIGs-LC + laser). The green fluorescence (very weak) of live cells was hardly observed in group of FIGs-LC + Laser; however, obvious green fluorescence remained in the group of FIGs + Laser despite many dead cells, indicating the best therapy potentials of FIGs-LC under laser irradiation. These results match well with the evaluations of ROS; the generated ROS causes high cytotoxicity on tumor cells and can be enhanced by LOx and CAT, suggesting that FIGs-LC can kill tumor cells effectively while sparing normal cells.

### In vivo cancer therapy and biosafety evaluation

The therapeutic performances of FIGs-LC were assessed on SMMC-7721 tumor-bearing BLAB/c nude mice. Prior to the in vivo tumor treatment, the passive targeting capability of FIGs-LC (at a dose of ICG of 6 mg/kg, 200 μL) was investigated by in vivo fluorescence imaging technology after intravenous administration. Obviously, strong fluorescence accumulations were detected on the tumor sites of mice with FIGs-LC injection at 8 h and exhibited a long-time tumor retention (Supplementary Fig. 28a), which might be ascribed to the inherent EPR effect of nano-sized FIGs-LC. As one of the FDA-approved NIR dye, ICG is clinically applied to evaluate the live function and blood flow, it is normal that most of free ICG were rapidly expelled through liver^34^. Finally, all mice were sacrificed, and their major organs and tumor tissues were dissected for semi-quantitative bio-distribution analyses; the obtained results also showed the effective and improved tumor retention of FIGs-LC (Supplementary Fig. 28b). Compared with the effective accumulation of FIGs-LC, no effective and long-time fluorescence accumulation appeared in the tumors of ICG-injected mice after 8 h. It is thus reasonable to conclude that the as-prepared FIGs-LC can serve as practical nanomedicines with passively targeting capability for cancer therapy.

**Fig. 6:**
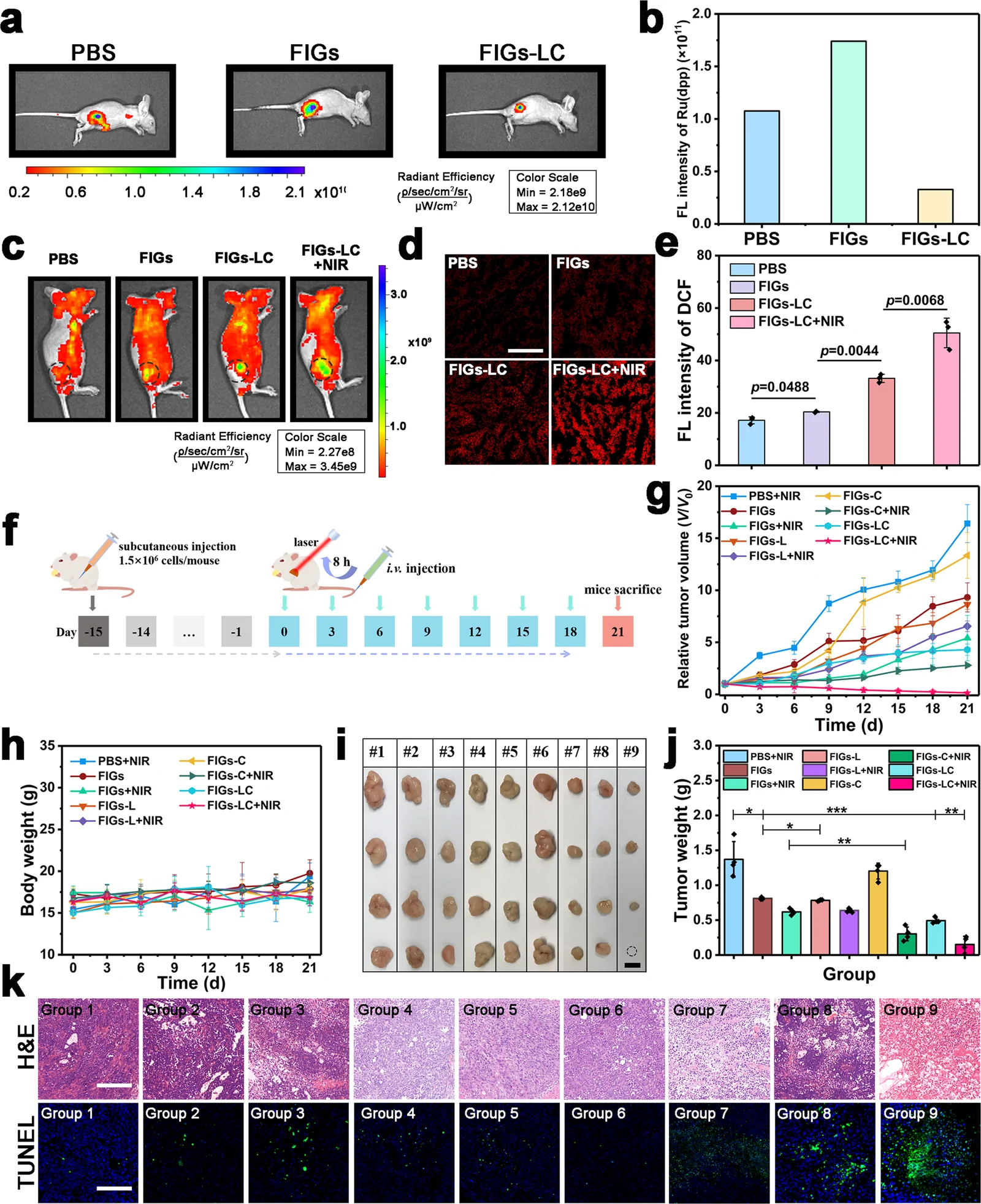
Therapeutic effect of FIGs-LC on the subcutaneous SMMC-7721 tumor mice model. **a** Intratumoral fluorescence imaging of O_2_ indicator Ru(dpp) after co-injected with different solutions into tumor tissues and **b** the relative semi-quantitative fluorescence intensity. **c** Intratumoral fluorescence imaging of ROS by DHE after co-injected with different solutions into tumor tissues and the NIR irradiation was conducted after 4 h. **d** DCFH-DA-stained tumor tissue sections from different groups at 12 h post-injection, scale bar: 200 μm. **e** Semi-quantitative fluorescence intensity of DCF. **f** Schematic illustration of in vivo treatment on tumor-bearing nude mice. Change in relative tumor volume (**g**) and body weight (**h**) during the treatment. **i** Digital photos of the excised tumors from the tumor-bearing mice after 21 days of treatment (1# (Group 1): PBS + NIR, 2# (Group 2): FIGs, 3# (Group 3): FIGs + NIR, 4# (Group4): FIGs-L, 5# (Group5): FIGs-L + NIR, 6# (Group 6): FIGs-C, 7# (Group7): FIGs-C + NIR, #8 (Group 8): FIGs-LC, #9 (Group 9): FIGs-LC + NIR), dot circle represents the tumor was eliminated completely, scale bar: 10 mm. **j** Average weight of excised tumors from the tumor-bearing mice after 21 days of treatment. **k** H&E staining and TUNEL staining images of tumor slices, scale bar: 200 μm. In **b**, data are presented as mean from two independent animal experiments. In **e**, **g**, **h**, **j**, data are presented as mean ± s.d. from three (**e**) or four (**g**, **h**, **j**) independent animal experiments, *p* values were analyzed by Student’s two-sided *t*-test (*p < 0.05, **p < 0.01, ***p < 0.001, n.s. represents no significant differences).

To verify the efficient generation and stability of O_2_ nanobubbles and therapeutic efficacy in the hypoxic TME, the in vivo fluorescence imaging of O_2_ and ROS on the subcutaneous SMMC-7721 tumor mice model has been conducted. As shown in Fig. 6a, b, the significantly decreased fluorescence signals were observed after being injected with FIGs-LC, which can be attributed to the increased detectable and accessible O_2_ inside the cells and tumors through the cascade biocatalytic conversion of CAT biocatalytic conversion of diffused low-concentration O_2_ molecules to the concentrated O_2_ micro/nanobubbles in the peptide-assembled hydrogel networks (Supplementary Fig. 16). The ROS generation in vivo was further evaluated by co-injecting ROS indicator dihydroethidium (DHE) and different solutions (PBS, FIGs, and FIGs-LC) into tumor tissues. As shown in Fig. 6c and Supplementary Fig. 29, as expected, the group of FIGs-LC plus NIR irradiation showed the highest ROS level. Furthermore, the DCFH-DA-stained pathological section analysis of tumor tissues from mice treated with different groups for the monitoring of ROS levels also confirmed that the FIGs-LC plus laser irradiation group showed the highest yield of ROS species (Fig. 6d, e), revealing its optimal therapeutic by the synergistic CDT and PDT in vivo.

Based on the sufficient accumulation capability and the satisfactory ROS generation within tumor tissues, the in vivo antitumor experiments were subsequently conducted on SMMC-7721 tumor-bearing BLAB/c nude mice (Fig. 6f). As anticipated, the tumors of mice that intravenously injected with FIGs-LC and received 808 nm laser irradiation were suppressed distinctly and showed an inhibition rate of 89.05%, which was higher than that of other control groups (PBS + NIR, FIGs, FIGs + NIR, FIGs-L, FIGs-L + NIR, FIGs-C, FIGs-C + NIR, FIGs-LC) due to the enzyme-enhanced ROS generation in the tumor region (Fig. 6g). In addition, the inhibition rates of other groups (FIGs, FIGs + NIR, FIGs-L, FIGs-L + NIR, FIGs-C, FIGs-C + NIR, FIGs-LC) that relative to the PBS control group were 40.88%, 54.93%, 40.51%, 53.28%, 12.23%, 77.92%, and 64.05%, respectively. It is noting that none of the mice exhibited a dramatical decrease in body weight during the entire treatment procedure (Fig. 6h), verifying that the administration of FIGs-LC is virtually harmless to the mice’s growth. After treating for 21 days, all tumor tissues were collected and weighted. The digital photographs and the average weight of tumors (Fig. 6i, j) further verified that FIGs-LC can safely and efficaciously inhibit the tumor growth with the assistance of NIR laser. Especially, there was individual tumor in the group of FIGs-LC disappeared completely, which was represented by a circle in the photograph (Fig. 6i). Noticeably, to eliminate the influence of photothermal effects from ICG molecules on the therapeutic results, the IR thermal images and the corresponding heating curves of mice injected with PBS, FIGs, FIGs-L, FIGs-C, and FIGs-LC irradiated with 808 nm laser (0.25 W) were conducted. As shown in Supplementary Fig. 30, the temperature changes at the tumor sites of mice in the laser irradiation process were monitored by an IR thermal imaging camera. After an initial temperature increase within ~120 s, the final tumor temperature increases were lower than 3 °C, which was not enough to induce significant harm to tumor tissues. In addition, the temperature decreases in some groups can be explained by the decrease of body temperature in the period of narcosis. Thus, in the present work, ICG is mainly used to produce ^1^O_2_ in PDT, while its photothermal effect on the therapeutic results is negligible.

To further confirm the therapeutic efficacy of FIGs-LC, both the hematoxylin and eosin (H&E) staining and terminal deoxynucleotidyl transferase-mediated dUTP nick end labeling (TUNEL) assay were conducted. Results demonstrated that the enzyme-enhanced ROS elevation led to severe cellular destruction and clear apoptosis to the tumor tissues (Fig. 6k). Specifically, the group of FIGs-LC plus NIR irradiation showed the highest green fluorescence of dead cells in the TUNEL images, indicating the extraordinary therapy outcomes of FIGs-LC. Additionally, the major organs (heart, liver, spleen, lung, and kidney) were excised and sliced for H&E staining (Supplementary Fig. 31); the intact cells in these images implied the desired biosafety and high biocompatibility of FIGs-LC. The biosafety and biocompatibility were further evaluated on healthy Kunming mice (6 weeks old). The pharmacokinetic profile of FRGs-LC was obtained after intravenous injection, the circulating half-life was calculated as 3.09 h, and the shifting time interval of the two elimination curves was 1.38 h (Supplementary Fig. 32). Then, the serum biochemical and hematological parameters of Kunming mice were assessed after intravenously injected with PBS and FIGs-LC. We found that the alanine aminotransferase, aspartate aminotransferase, and alkaline phosphatase levels of both groups were within normal ranges, validating that the administration of FIGs-LC did not damage the physiological function of livers (Supplementary Fig. 33a). Meanwhile, the normal and healthy status of kidneys were confirmed by the levels of creatinine (Crea) and urea (Supplementary Fig. 33b). In addition, the comparisons between the control group and treated group showed that no prominent fluctuation was found in the blood indexes, including white blood cells, red blood cells, hemoglobin, red blood cell volume, mean corpuscular volume, mean corpuscular hemoglobin, etc., indicating the normal biochemical status during the whole assessment period (Supplementary Table 2). These results comprehensively confirmed the effective therapy capability and the high biocompatibility of FIGs-LC for tumor treatment.

## Discussion

Bioinspired by the H_2_O_2_ activation reaction and the O_2_ redistribution pathways of peroxisome, the enzyme complex (LOx and CAT)-loaded hybrid nanogel system (FIGs-LC) to achieve ROS regulation has been proposed. Firstly, the LOx-modulated CDT can be fulfilled by catalyzing endogenous lactate to produce H_2_O_2_, and further reacting with the GSH-activable Fe^2^^+^ ions to produce the ·OH; simultaneously, the CAT can cascade decompose the H_2_O_2_ into O_2_, which immediately accept energy from the excited-state ICG molecules close to it and are converted to ^1^O_2_ under an 808 nm laser irradiation, realizing synchronous O_2_ supply and ^1^O_2_ generation for PDT. By fully considering the regulation of LOx and CAT enzyme complex, the reactions of FIGs-LC can be regulated to significantly elevate the intracellular and TME ROS level. In vitro cytotoxicity tests showed that the inhibition efficacy of cancer cell proliferation was significantly promoted by the enzyme-amplified ROS production (FIGs-LC). In vivo antitumor experiments were performed on tumor-bearing nude mice, and the results further verified the FIGs-LC inhibited the tumor growth (inhibition rate: 89.05%) and exhibited negligible side effects during the treatment procedure. Concurrently, FIGs-LC exhibited no distinct harm to the normal tissues/organs at the same dosages due to the great distinctions from the TME, indicating their good biosafety and biocompatibility. Besides, histopathology analyses and serum biochemical and hematological parameters tests demonstrated that the FIGs-LC induced significant cell apoptosis and did not destroy the major organs. Both the facile design of hybrid structure with micelle assembly/organosilica framework/supramolecular network, and the ROS regulation can potentially guide the synthesis and therapeutic research of bioinspired ROS regulator for treating tumors of different redox state, size, and subcutaneous depth in the further applications.

## Methods

### Materials and reagents

Reagents include MPTMS (95%), tetrahydrofuran (THF), ammonium hydroxide aqueous solution (28%), 1-ethyl-3-(3-dimethylamino propyl) carbodiimide hydrochloride (EDC), *N*-hydroxysulfosuccinimide sodium salt (Sulfo-NHS), catalase (CAT), and LOx were purchased from Sigma-Aldrich. Indocyanine Green (ICG, 98%) was purchased from Hwrk Chem. Acid phosphatase (AP), 1,2-dedecanediol was purchased from TCI (Shanghai) Development Co., Ltd., and 2-[4-(2-hydroxyethyl) piperazin-1-yl] ethanesulfonic acid (HEPES), tris(hydroxymethyl) aminoethane (Tris), and Fmoc-Tyr (H_2_PO_3_)-OH were purchased from J&K Scientific (China). SOSG was purchased from Dalian Meilun Biotechnology Co., Ltd. Methylene blue (MB), hydrogen peroxide (H_2_O_2_), glutathione (GSH), oleic acid, oleylamine, biphenyl ether, *n*-hexane, 1,10-phenanthroline monohydrate, and lactate (90% in water) were purchased from Aladdin. The 5,5-dimethyl-1-pyrroline *N*-oxide (DMPO) was purchased from Nantong Feiyu Biological Technology Co., Ltd. FBS was purchased from Zhejiang Tianhang Biotechnology Co., Ltd, China. DHE and cell culture products were purchased from Keygentec (Nanjing, China). All the reagents, except to that were not pointed out specially, were analytical grade, and ultra-pure water was used in the preparation of all samples.

### Characterization and apparatus

The hydrodynamic sizes and zeta potentials of NPs were measured at 25 °C with a Malvern Zeta-sizer Nano Series. FT-IR spectra were recorded with a Thermo Scientific Nicolet 6700. Raman shift spectra were recorded with a Renishaw Invia Reflex Laser Micro-Raman Spectrometer with 785 nm laser. TEM images were taken by a JEM 2100F electron microscope operated at 200 kV and a JEM 1400 electron microscope operated at 100 kV. Emission spectra were recorded with a Shimadzu Fluorescence Spectrophotometer RF-5301PC (1 cm quartz cell). FC was conducted by BD Accuri C6. CLSM images were taken by Nikon A1 Confocal Laser Scanning Microscopy (CLSM). UV-Vis-NIR spectra were measured with a Shimadzu spectrophotometer UV-3600 (1 cm quartz cell). Thermogravimetric analyses (TG) were conducted on a thermal analyzer (PerkinElmer TGA 8000). Electron spin resonance was measured with a Bruker EMX-8/2.7 (100G-18 KG) spectrometer. Nanobubbles were tested by the NTA (Claire Hannell; NanoSight Limited, Salisbury, UK).

### Synthesis of block copolymer

The amphiphilic block copolymer poly (styrene)_110_-*b*-poly (acrylic acid)_16_ (PS_110_-*b*-PAA_16_) was synthesized via sequential atomic transfer radical polymerization (ATRP) method^35^. Firstly, the mixture with 4.19 g of Cu(I)Br, 81 mL of *t-*butyl acrylate, 3.67 mL of methyl-2-bromopropionate, 6.83 mL of PMDETA, and 100 mL of dry toluene reacted at 60 °C under nitrogen flow for 6 h after eliminating the gas, and was quenched in liquid nitrogen. The solvent was evaporated to yield viscous polymer oil after removing the nantokite. Finally, after drying under vacuum for 24 h, the poly (*t*-butyl acrylate)_16_ (PtBA_16_, M_n_ = 2090, M_w_/M_n_ = 1.16) was synthesized.

To obtain P*t*BA-*b*-PS, the mixture with 0.63 g of CuBr, 9 g of P*t*BA, 54.4 mL of styrene, 1.79 mL of PMDETA and 56 mL of dry toluene were reacted for 6 h at 90 °C after eliminating the gas by nitrogen flow and then quenched the polymerization in liquid nitrogen. Then, the white solid was obtained by removing nantokite, cold methanol precipitation, and filtration. Finally, after drying under vacuum for 24 h, the product P*t*BA_16_*-b-*PS_110_ (M_n_ = 13,600, M_w_/M_n_ = 1.10) was synthesized.

For the hydrolysis of P*t*BA-*b*-PS to PS-*b*-PAA, 30 g of P*t*BA*-b-*PS and 2.7 mL of trifluoroacetic acid were dissolved in 200 mL of dichlormethane and stirred for 24 h. Then, the white solid was obtained by cold methanol precipitation and filtration. Finally, after drying under vacuum for 24 h, the PAA_16_*-b-*PS_110_ was synthesized.

### Synthesis of Fe_3_O_4_

The hydrophobic 6 nm Fe_3_O_4_ NPs were synthesized by the report of Sun et al.^36^. Briefly, iron (III) acetylacetonate (4.0 mmol) and 1,2-dedecanediol (22.3 mmol) were dissolved in the mixture of biphenyl ether (40.5 mL), oleic acid (12.2 mmol), and oleylamine (17.2 mmol). Then the solution was heated to 200 °C under N_2_ atmosphere for 2 h and followed a reflux at 300 °C for 1 h. The Fe_3_O_4_ NPs were separated by centrifugation (4000*g*, 6 min), and re-dispersed in *n*-hexane (10 mL, containing 0.5 mL of oleic acid and 0.5 mL of oleylamine). After centrifugation (7200*g*, 6 min), the supernatant was collected into 50 mL of absolute ethanol and centrifugated at 4000*g* for 6 min. The Fe_3_O_4_ precipitation was obtained and dried in vacuum at 60 °C overnight, and re-dispersed in THF (25 mg/mL) for subsequent use.

### Synthesis of Fe_3_O_4_@IHPs

The synthesis of hybrid micelles was based on our previous works^37,38^. Briefly, PS_110_-*b*-PAA_16_ (20 mg) and Fe_3_O_4_ NPs (15 mg) were dissolved in THF (10 mL) in a glass breaker (150 mL) and treated in an ultrasonic cleaner for 6 min. Ultra-pure water 40 mL was quickly poured into the THF solution under vigorous stir. The resulted micellar solution was immersed in a dialysis bag (molecular weight cutoff (MWCO) = 12,600 Da) against deionized water for 24 h to eliminate THF. Then NH_3_·H_2_O (1 mL) and ICG (4 mg) were added into the micellar solution, and MPTMS (50 μL) was dropwise added into the homogeneous solution. The reaction was continued for 24 h. The NH_4_OH and unencapsulated ICG molecules were eliminated via dialysis for 2 days.

### Synthesis of Fe_3_O_4_@IHPs@nanogels (FIGs)

The Fe_3_O_4_-encapsulated hybrid NPs were centrifuged (19,000*g*, 20 min, three times) from solution and resuspended in HEPES buffer (40 mL, 0.1 M). Carboxy groups of PAA blocks were activated by EDC (10 mg) and Sulfo-NHS (10 mg) for 4 h. After washing three times with ultra-pure water, AP (15 mg) was added into the solution and reacted for 12 h. Then, the NPs were washed with water three times and resuspended into ultra-pure water (30 mL). To prepared nanogels, Fmoc-Tyr (H_2_PO_3_)-OH solution (10 mL, 2 mg/mL, contain 0.2% Na_2_CO_3_) was poured into the acquired solution and stirred for 24 h at room temperature. The FIGs were then separated from the solution via the centrifugation (19,000*g*, 20 min) and washed three times with deionized water, and then resuspended in Tris·HCl buffer (40 mL, 1.0 M, pH 6.0).

### Synthesis of FIGs-LC (immobilization of LOx and CAT)

LOx (20.5 U) was added into the FIG solution (10 mg) and stirred at room temperature for 12 h. The residual unreacted LOx was then separated from the NPs by centrifugation and then washed with deionized water for three times. CAT (41.0 U) was added into the obtained FIGs-L NPs and stirred at room temperature for 12 h. The residual unreacted CAT was then separated from the NPs by centrifugation and then washed with deionized water for three times.

FIGs-LC with different ratios between LOx and CAT was prepared as follows. The mixture of a: LOx (41.0 U) and CAT (20.5 U) (LOx:CAT = 2:1), b: LOx (20.5 U) and CAT (41.0 U) (LOx:CAT = 1:2), c: LOx (15.375 U) and CAT (46.125 U) (LOx:CAT = 1:3) was added into the FIG solution (10 mg), respectively, for the three groups and stirred at room temperature for 12 h. Finally, the LOx/CAT co-loaded NPs (FIGs-LC) were obtained by centrifugation. The synthesis of control sample IGs-LC without Fe_3_O_4_ NPs was similar with the FIGs-LC, except for the absence of Fe_3_O_4_ NPs in the initial micellar solution.

### Loading amount and activity test of LOx and CAT

The supernatant and the washed solution were kept for determination of enzyme concentrations by Bradford method. LOx activity was measured using a peroxidase-coupled spectrophotometric assay (Sigma) in which the H_2_O_2_ produced in the LOx reaction is further reacted with peroxidase in the presence of 4-aminoanipyrine and *N*,*N*-dimethylaniline to give a quinonediimine dye that is detected. About 5–10 μL of enzyme appropriately diluted in 50 mM potassium phosphate buffer, pH 7.0, was added to a solution containing 40 mM 3,3-dimethylglutaric acid, 2.5 units peroxidase, 1.5 mM 4-aminoantipyrine, 50 mM lactate, and 0.04% (v/v) *N*,*N*-dimethylaniline in a total volume of 0.5 mL. The pH of the solution was pH 6.0. After a reaction time of between 10 min at 37 °C, 1 mL of 0.25% (w/v) dodecylbenzenesulfonic acid was added to stop the reaction. The produced quinonediimine dye was measured spectrophotometrically at 565 nm with a Varian Cary 50 Bio UV-visible spectrophotometer at 25 °C.

The activity of immobilized CAT was determined in the system with FIGs-LC (10 mM potassium phosphate buffer, pH = 6.0, with 0.45 U/mL CAT loaded) and H_2_O_2_ solution (0.067 M) at the absorbance of 240 nm at 25 °C. The activity of the free CAT was also tested as a control by adding CAT solution at the same enzyme concentration found in FIGs-LC. The specific activity is expressed as the activity of FIGs-LC relative to the activity of free CAT:CAT_activity_ (%) = *D* × 100/*D*_Free_, where *D* and *D*_Free_ are the H_2_O_2_ degradation rates (the slope of the absorbance curve) of FIGs-LC and the free CAT, respectively.

The activities of LOx and CAT in FIGs-LC at different pH (pH = 7.4, 6.0) environment were further tested according to the above methods. Moreover, the storage activities of them in FIGs-LC were also performed during 28 days at different time interval.

### Stability and GSH-sensitivity of FIGs-LC

FIGs (0.05 mg/mL) were incubated in different conditions (water, culture medium, PBS (10% FBS), PBS (10% FBS + 1.0 mM GSH), PBS (10% FBS + 5.0 mM GSH), PBS (10% FBS + 10.0 mM GSH)) at 37 °C, the sizes of NPs at given time points were measured by DLS. Morphologies of FIGs were obtained by TEM. FT-IR and Ramen spectra of FIGs-LC were recorded before and after treated with 10.0 mM GSH as well. To evaluate the improved ICG stability after doped into organosilica framework, free ICG and FIGs-LC (4 μg/mL) were incubated in PBS (containing 10% FBS) at 37 °C for 5 days, and absorbance at 780 nm was measured by UV-Vis spectrophotometer.

### Release behavior of Fe ions from FIGs-LC

FIGs-LC (1.0 mL, Fe_3_O_4_ concentration was 100 μg/mL) was added into 4.5 mL of PBS (containing 10 mM GSH and 100 μM H_2_O_2_, and pH was adjusted to 6.0 by adding 1 M NaOH) and shaken at 37 °C. At predetermined time points, 1.0 mL of the solution was taken out and refreshed by 1.0 mL of fresh buffer. Then 1,10-phenanthroline monohydrate (2.0 mL, 1.0 mg/mL) was added into the taken solution and measured the absorbance at 510 nm by UV-Vis spectrophotometer. The cumulative iron release was calculated through the standard Fe^2^^+^ concentration–absorbance curve.

### Degradation of MB

Methylene blue (MB) can be degraded by hydroxyl radicals (·OH), which was used to assess the production of ·OH from Fenton reaction. PBS with 20 μM of GSH and 0.5 mM of lactate was applied to simulate the normal physiological environment, PBS (pH 6.0) with 10 mM of GSH, 2 mM of lactate and of 100 μM of H_2_O_2_ was used to simulate the TME. MB (100 μL, 1.26 mM) was added into the different PBS (2.90 mL) and incubated at a 37 °C shaker. To evaluate the Fenton reaction under different conditions, the absorbance at 664 nm of MB was recorded by UV-Vis spectrophotometer after centrifugation at predetermined time points (0, 3, 12, 24 h).

The MB relative degradation rate was calculated by the formula:

1 $${{{{{\rm{Degradation}}}}}}\,{{{{{\rm{rate}}}}}}\,({{{{{{\rm{h}}}}}}}^{-1})=\frac{{M}_{0}-{M}_{{{{{{\rm{t}}}}}}}}{{M}_{{{{{{\rm{Fe}}}}}_{{3}{{{{{\rm{O}}}}}}_{4}}}}\cdot \Delta t}$$

*M*_0_ is the concentration of MB in the initial solution, *M*_t_ is the concentration of MB in solution at predetermined time points; MB concentration is calculated by the standard curve; $M_{{{{{{{{\rm{Fe}}}}}}}_{3}{{{{{\rm{O}}}}}_{4}}}}$ is the concentration of the Fe_3_O_4_; ∆*t* is the time interval.

### In vitro·OH detection by ESR spectroscopy

Hydroxyl radicals (·OH) generated by Fenton reaction were detected by ESR measurements with a Bruker EMX-8/2.7 (100G-18 KG) spectrometer at room temperature. 5,5-Dimethyl-1-pyrroline-*N*-oxide (DMPO) was used as a spin trapper for the detection of ·OH. Ten microliters of DMPO was added into 1 mL of different solutions that were incubated at 37 °C for 6 h (Fe_3_O_4_ concentration is 15 μg/mL) and filtrated through a 0.22 µm NY membrane, and detected immediately. Besides, 10 μL of DMPO was added into 1.0 mL of PBS, which was arranged as a control group. The settings of ESR measurement parameters were as follows: 6.42 mW microwave power, 9.89 GHz microwave frequency, 1.00G modulation amplitude, 100.00 kHz modulation frequency.

### Detection of oxygen production by FIGs-LC

Ten microliters of FIGs or FIGs-LC (100 μg/mL), 100 μL of lactate (2 mM, pH was adjusted to 7.0 by NaOH), 100 μL of H_2_O_2_ (100 μM), 10 μL of Tris (4, 7-diphenyl-1,10-phenanthroline) Ruthenium (II) dichloride (Ru(dpp), 1.0 mg/mL in DMF), and 2.78 mL of PBS (pH 6.0) were added to the quartz cuvette. The fluorescence spectra of Ru (dpp) were measured by a fluorescence spectrophotometer (Ex = 470 nm. Em = 607 nm); the increased O_2_ concentration in solutions was reflected by the quenching of fluorescence.

### In vitro measurement of ^1^O_2_ generation

Fifteen microliters of SOSG solution (1 mM in methanol) was added to the sample solutions (ICG concentration is 8 μg/mL), then irradiated by an 808 nm laser (0.25 W) for 0, 2, 5, 10 min, and the fluorescence of SOSG (Ex = 504 nm, Em = 525 nm) was recorded after irradiation. For ESR measurement, 2,2,6,6-tetramethylpiperidine (TEMP) was used as a spin trapper for the detection of ^1^O_2_. In all, 5.6 mM TEMP was used in the sample solutions, after irradiation by 808 nm laser and filtrated through a 0.22 µm NY membrane, the measurements were carried out.

### Cellular uptake of FRGs-LC

Human hepatocellular carcinoma SMMC-7721 cells were purchased from Jiangsu Keygen Biotech Corp., Ltd. RhB-loaded FRGs-LC were used for cellular uptake. SMMC-7721 cells were seeded in six-well plates at a density of 1.0 × 10^5^ cells/well, and incubated overnight, and then the culture medium was replaced by fresh medium containing FRGs-LC (0.2 mg/mL), and incubated for the different time (0, 2, 4, 8, 12, and 24 h). After incubation, the cells were washed and collected in 0.5 mL of PBS, and the fluorescent intensities were analyzed with a flow cytometer.

### Intracellular ROS detection

For the determinations of intracellular ^1^O_2_ generation, SMMC-7721 cells were seeded on glass-bottom culture dishes at a density of 1.0 × 10^5^ cells per well. The FIGs and FIGs-LC (with ICG concentration of 4.0 μg/mL) were incubated with cells for 6 h. Especially, lactate (2.0 mM)/H_2_O_2_ (100 μM)-containing medium was used in cell culture. Then the medium was replaced by a serum-free medium with 2.0 μM of SOSG. After incubated for another 30 min, the cells were washed with PBS and irradiated with an 808 nm laser (0.25 W) or shielded in dark. The cells were then observed by CLSM.

The intracellular ROS production was also evaluated, and SMMC-7721 cells were seeded on glass-bottom culture dishes at a density of 1.0 × 10^5^ cells/well. Then the medium was replaced with ICG-, FIGs-, and FIGs-LC-containing culture medium (with ICG concentration of 4.0 μg/mL), and incubated for 12 h. Specially, lactate (2.0 mM)/ H_2_O_2_(100 μM)-containing medium was used in cell culture. Then the medium was replaced by a serum-free medium with 10 mM of DCFH-DA. After incubated for another 20 min, the cells were washed with PBS and irradiated with an 808 nm laser (0.25 W) or shielded in dark. The cells were then observed by CLSM.

### Intracellular NADH/NAD^+^measurement

The concentration of NADH and NAD^+^ and NADH/NAD^+^ were measured using NAD^+^/NADH Assay Kit with WST-8 (Beyotime Biotechnology). Specifically, SMMC-7721 cells were seeded on six-well plates (1.0 × 10^6^ cells/well) and incubated for overnight. Then, the medium was replaced by fresh medium containing FIGs, FIGs-L, or FIGs-LC (0.3 mg/mL). After incubation for 4 h, the cells were washed and 200 μL of extracting solution was added. Finally, the concentration of NADH or NAD^+^ was measure by a multiplate reader.

### Cytotoxicity measurements

SMMC-7721 cells and mouse embryo fibroblast (NIH-3T3) cells were purchased from Jiangsu Keygen Biotech Corp., Ltd. The cell counting method was used to examine the cell viability, which avoided the errors in MTT or CCK-8 tests due to the interference of the reducibility of disulfide bonds and strong absorbance of ICG in the UV-Vis spectrum. Specifically, NIH-3T3 or SMMC-7721 cells were seeded on 24-well plates (5.0 × 10^4^ cells/well), and incubated for overnight. Then, the medium was replaced by fresh medium containing FIGs, FIGs-L, or FIGs-LC at different concentrations (Fe_3_O_4_ concentration: 0, 0.63, 1.25, 2.50, 5.00, 10.00 μg/mL, respectively) for 12 h, followed by the irradiation of an 808 nm laser (0.25 W, 5 min) or shielded in dark. After incubated for another 36 h, the cells were trypsinized, and 100 μL of cell suspension and 100 μL of trypan blue solution (4.0 mg/mL) was mixed for 5 min. The cells were counted using an optical microscope (performed each concentration five times), the brilliant blue cells represent dead cells and unstained cells represent living cells, and the cell viability was calculated by the following equation:

2 $${{{{{\rm{Cell}}}}}}\,{{{{{\rm{viability}}}}}}( \% )=\frac{{{{{{\rm{Count}}}}}}\,{{{{{\rm{of}}}}}}\,{{{{{\rm{live}}}}}}\,{{{{{\rm{cells}}}}}}\,{{{{{\rm{of}}}}}}\,{{{{{\rm{treated}}}}}}\,{{{{{\rm{groups}}}}}}}{{{{{{\rm{Count}}}}}}\,{{{{{\rm{of}}}}}}\,{{{{{\rm{live}}}}}}\,{{{{{\rm{cells}}}}}}\,{{{{{\rm{of}}}}}}\,{{{{{\rm{control}}}}}}\,{{{{{\rm{group}}}}}}}\times 100$$

### Cell apoptosis analysis

SMMC-7721 cells were incubated with FIGs or FIGs-LC (at the Fe_3_O_4_ concentration of 10 μg/mL) for 12 h, then irradiated with an 808 nm laser (0.25 W) for 5 min, and incubated for another 36 h. Following the incubation with 5 μL of annexin V-FITC and PI at room temperature for 15 min. Finally, the cells were washed and trypsinized for FC analysis. The apoptosis analysis for IGs and IGs-LC (0.3 mg/mL) were conducted with similar protocols except for the 24 h incubation time.

### Animal experiments

With the approval of Animal Ethics Committee of East China University of Science and Technology, and under the Guidance for Care and Use of Laboratory Animals of East China University of Science and Technology, animal experiments were carried out. All mice were housed in a specific pathogen-free environment at 26 ± 1 °C and 50 ± 5% humidity, with a 12 h light–dark cycle.

### Pharmacokinetics

Female Kunming mice (*n* = 3, 6-week-old) were intravenously injected with FRGs-LC. At predetermined time points (2, 8, 15, 30 min, 1, 2, 4, 8, 24 h), 5 μL of blood was drawn from the tail vein and dispersed into 495 μL of heparin sodium-containing physiological saline (50 U/mL). The fluorescent intensities at 594 nm were recorded by a fluorescence spectrophotometer. The circulating half-live of FRGs-LC was calculated by a double-compartment pharmacokinetic model. The in vivo eliminating rate curves of FRGs-LC were conducted by plotting ln (*I*_594_) against time and fitted according to the two-compartment models.

### Tumor mouse model

To establish tumor models, 100 μL of SMMC-7721 cells (1.5 × 10^6^ cells) suspension was subcutaneously injected into the right armpit of female nude mice (6 weeks old). Treatments were carried out when tumor volumes reached approximately 100 mm^3^.

### In vivo fluorescence imaging

SMMC-7721 tumor-bearing nude mice were intravenously injected with ICG or FIGs-LC (at the dosage of ICG of 6 mg/kg), after which fluorescence was observed at 2, 4, and 8 h. Finally, the mice were sacrificed, the tumor tissues and major organs were excised, and observed by an imaging system.

### Intratumoral oxygen fluorescence imaging

Before the fluorescence imaging, all mice were fasted 8 h. Ten microliters of PBS or FIGs or FIGs-LC (2.5 mg/mL, containing 0.5 mg/mL of Ru(dpp)) were injected into the tumor tissues, and the mice were placed in the dark cages for 4 h. Fluorescence images were then collected by an imaging system (IVIS Spectrum, PerkinElmer).

### Intratumoral ROS fluorescence imaging

Before the fluorescence imaging, all mice were fasted 8 h. Ten microliters of PBS or FIGs or FIGs-LC (2.5 mg/mL, containing 0.5 mg/mL of DHE) were injected into the tumor tissues. The laser irradiation (0.25 W, 5 min) was conducted after 4 h; all the mice were placed in the dark cages before fluorescence imaging. Fluorescence images were collected by an imaging system after 12 h post-injection. For the dichloro-dihydro-fluorescein diacetate (DCFH-DA) stained pathological section analyses, the mice were sacrificed at 24 h post-injection, and their tumor tissues were dissected and immediately frozen in a refrigerator (−60 °C) for making sections. Then the DCFH-DA assay were used for ROS staining of tumor tissues.

### In vivo antitumor studies

Tumor-bearing nude mice were randomly divided into nine groups (*n* = 4), and intravenously injected with PBS, FIGs, FIGs-L, FIGs-C, and FIGs-LC (with Fe_3_O_4_ content of 10 mg/kg, ICG content of 5.5 mg/kg) every 3 days for seven times, respectively. After an 8-h interval, the tumors of five groups (PBS + NIR, FIGs + NIR, FIGs-L + NIR, FIGs-C + NIR, and FIGs-LC + NIR) were exposed to NIR laser irradiation (808 nm, 0.25 W) for 5 min or not. The tumor sizes were measured with a caliper every 3 days and tumor volumes were calculated by the formula: *V* (mm^3^) = *L* × *W*^2^/2 (*L* is the largest diameter and *W* is the smallest diameter). After 21 days of treatment, all mice were sacrificed and their tumor tissues were segregated and weighed. The tumor inhibition ratio was calculated by the formula:

3 $${{{{{\rm{The}}}}}}\,{{{{{\rm{inhibition}}}}}}\,{{{{{\rm{ratio}}}}}}( \% )=\,\frac{{m}_{{{{{{\rm{control}}}}}}}-{m}_{{{{{{\rm{treated}}}}}}}}{{m}_{{{{{{\rm{control}}}}}}}}\times 100$$

*m*_control_ was the average tumor weight of the control groups and *m*_treated_ was the average tumor weight of the treated groups.

### Pathological evaluations

For histological analysis, mice were sacrificed after the treatment, and tumor tissues in each group were separated. The tumor tissues were dissected to make paraffin sections for further H&E staining and TUNEL staining assays. Images of TUNEL staining assay were obtained under a magnification of 20 and that of H&E were obtained under magnification of 20. To evaluate the systemic toxicity, the major organs were also dissected to make paraffin sections for further H&E staining.

For in vivo biosafety evaluations, female Kunming mice (6 weeks old) were randomly separated into two groups (*n* = 3). PBS, FIGs-LC were intravenously injected into the mice; all Kunming mice were sacrificed for blood routine and biochemistry test after 21 days.

### Statistical analysis

Microsoft Excel 2019, GraphPad prism 8, Origin 2017, Flowjo_V10, and Image J 1.52p were used for data processing or statistical analysis. Data in manuscript and Supplementary Information are presented as mean ± standard deviation. Student’s *t*-test was carried out for statistical significance. *p < 0.05 is considered statistically significant, **p < 0.01 and ***p < 0.001 are considered to indicate extreme significance, n.s. represents no significant.

### Reporting summary

Further information on research design is available in the Nature Research Reporting Summary linked to this article.

## Supplementary information

Supplementary information

Reporting Summary

## Source data

Source Data

## Data Availability

The experimental data supporting the findings of this study are available within the article, Supplementary Information, and Source Data. Additional data are available from the corresponding authors upon reasonable request. Source data are provided with this paper.
