# Supplementary material for: Peroxisome inspired hybrid enzyme nanogels for chemodynamic and photodynamic therapy
Source: Nat Commun. 2021 Sep 2;12:5243. doi: 10.1038/s41467-021-25561-z (PMC8413279; doi:10.1038/s41467-021-25561-z)
Supplement: Supplementary file 1 — Supplementary information [file 41467_2021_25561_MOESM1_ESM.pdf]

Supplementary Information for

Peroxisome inspired hybrid enzyme nanogels for chemodynamic and photodynamic therapy

Qin et al

List of supplementary figures and tables

Supplementary Figure 1. Schematic diagram of enzymatic manipulation in the extracellular lactate: pyruvate ratio.
Supplementary Figure 2. TEM and SEM images of the Fe_3O_4@IHPs and FIGs.
Supplementary Figure 3. Digital photographs of the synthesized macro-hydrogel.
Supplementary Figure 4. The particle distribution under Brownian motion measured by NTA.
Supplementary Figure 5. Variations of Zeta potentials in the synthesis process of FIGs-LC.
Supplementary Figure 6. Enzymatic activity under different pH values and the storage activity.
Supplementary Figure 7. The cumulative release of enzyme of FIGs-LC.
Supplementary Figure 8. Stability of free ICG and FIGs-LC.
Supplementary Figure 9. UV-Vis-NIR absorption spectra of ICG and FIGs-LC.
Supplementary Figure 10. TEM images of the degradation of FIGs-LC.
Supplementary Figure 11. GSH-responsive size changes of FIGs-LC.
Supplementary Figure 12. The standard concentration-absorbance curve of Fe^{2+} solutions.
Supplementary Figure 13. The Fe^{2+} release under normal physiological conditions.
Supplementary Figure 14. The MB degradation curve under normal physiological conditions.
Supplementary Figure 15. O_2 nanobubble distributions under Brownian motion in different solutions.
Supplementary Figure 16. High density of O_2 in the micro/nanobubbles by STXM imaging.
Supplementary Figure 17. Temperature elevation of FIGs-LC solutions under laser irradiation.
Supplementary Figure 18. The standard concentration-absorbance curve of MB solutions.
Supplementary Figure 19. Fluorescence emission spectrum of FRGs-LC.
Supplementary Figure 20. Flow cytometry analysis of SMMC-7721 after incubation with FRGs-LC.
Supplementary Figure 21. CLSM images of cellular uptake of SMMC-7721 cancer cells incubated with FRGs-LC.
Supplementary Figure 22. Analysis of intracellular NADH and NAD^+.
Supplementary Figure 23. The cell apoptosis after co-incubation with IGs and IGs-LC
Supplementary Figure 24. Confocal images of the intracellular O_2 level.
Supplementary Figure 25. CLSM images for intracellular ROS detection.
Supplementary Figure 26. The cytotoxicity of FIGs-L co-incubation with normal cells and cancer cells.
Supplementary Figure 27. Gating strategy to analyze the apoptosis of cells in Fig. 5h-k.

Supplementary Figure 28. In vivo and ex vivo fluorescence images of tumor-bearing mice at different time after intravenous injection of ICG or FIGs-LC.
Supplementary Figure 29. The semi-quantitative fluorescence of ROS indicator in tumors after co-injected with different solutions.
Supplementary Figure 30. IR thermal imaging of mice during the therapy process.
Supplementary Figure 31. H&E staining images of the major organs (heart, liver, spleen, lung, and kidney).
Supplementary Figure 32. Pharmacokinetics of FRGs-LC.
Supplementary Figure 33. Blood biochemistry assays.
Supplementary Table 1. The loading efficiency and loading amount of LOx and CAT.
Supplementary Table 2. Hematology analysis of mice with FIGs-LC administration.

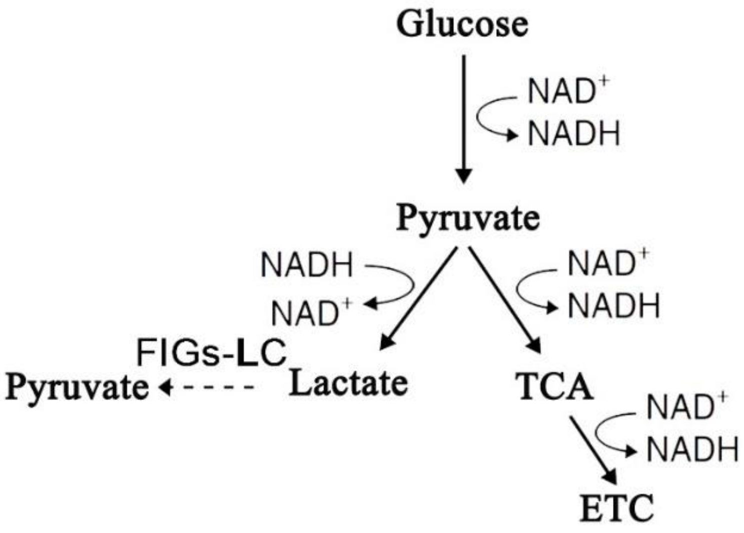

Supplementary Figure 1. Schematic diagram of enzymatic manipulation in the extracellular lactate: pyruvate ratio to impact the cytoplasmic NADH: NAD^+ ratio by FIGs-LC (TCA cycle: tricarboxylic acid cycle, ETC: electron transport chain).

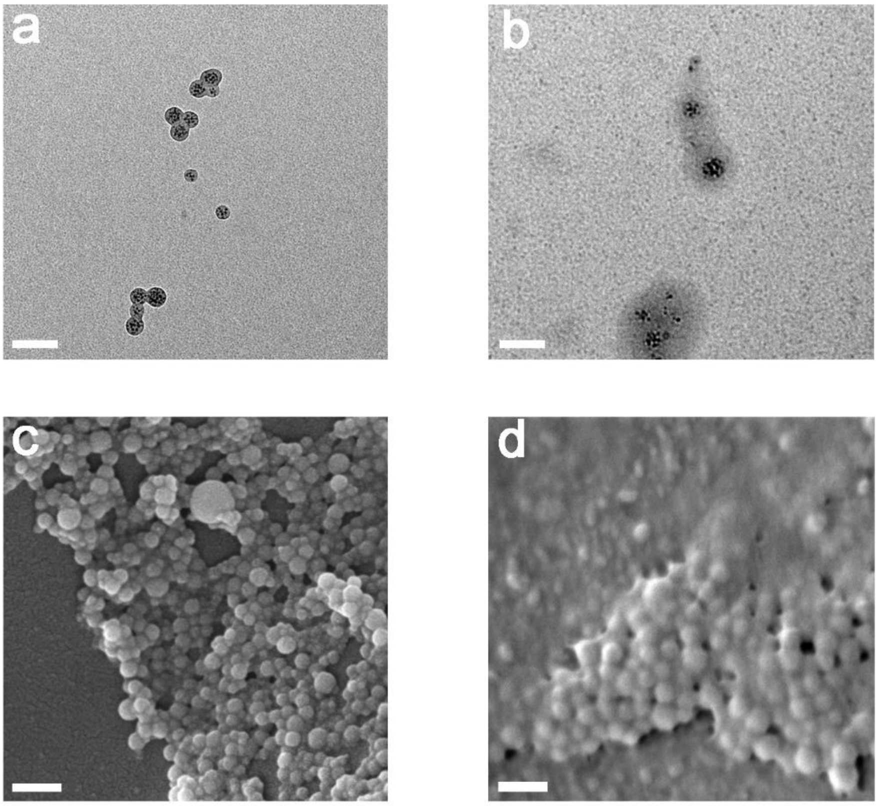

Supplementary Figure 2. TEM images of the Fe_3O_4@IHPs (**a**) and FIGs (**b**) and SEM images of the Fe_3O_4@IHPs (**c**) and FIGs (**d**), scale bar: 100 nm.

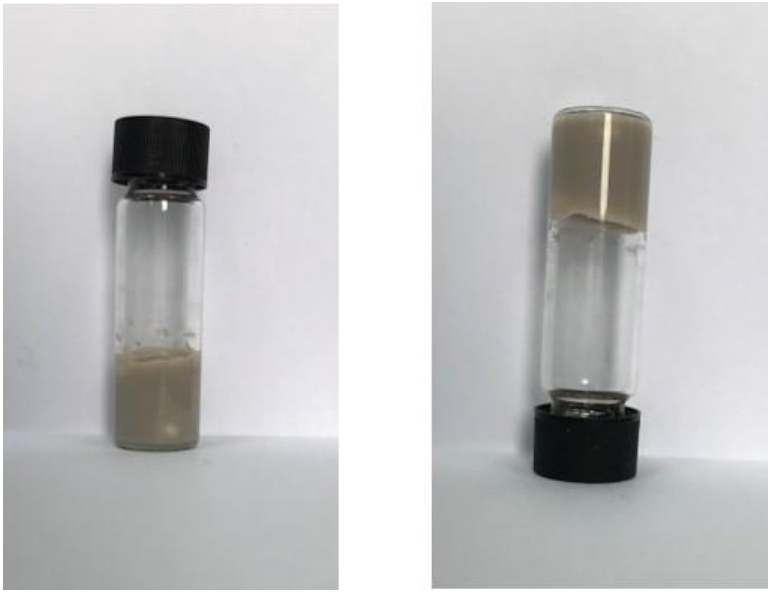

Supplementary Figure 3. The formation of the macro-hydrogel containing Fe_3O_4@IHPs after increasing the feeding amount of AP and Fmoc-Tyr (H_2PO_3)-OH.

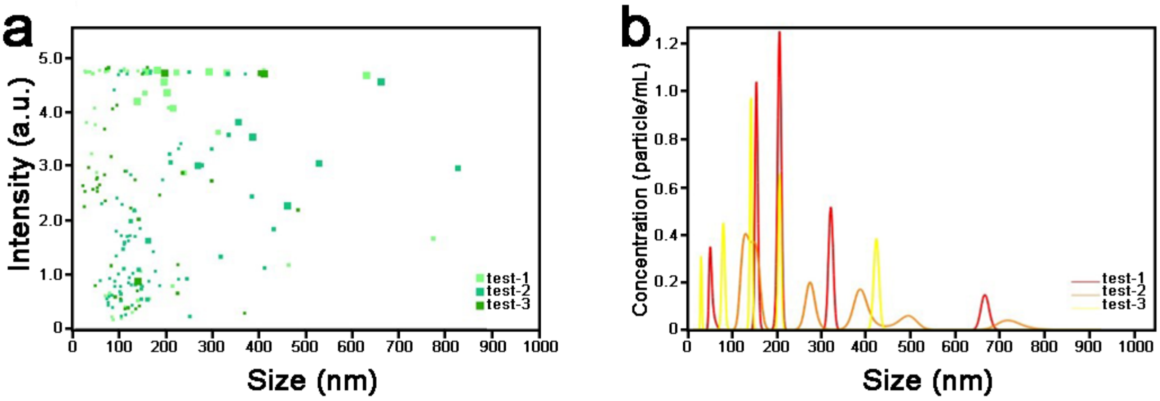

Supplementary Figure 4. The image of particle distribution (**a**) under Brownian motion and the corresponding size distribution (**b**) of FIGs-LC tested by the NanoSight technology analysis (NTA). Test-1, test-2, and test-3 represent three independent replicates.

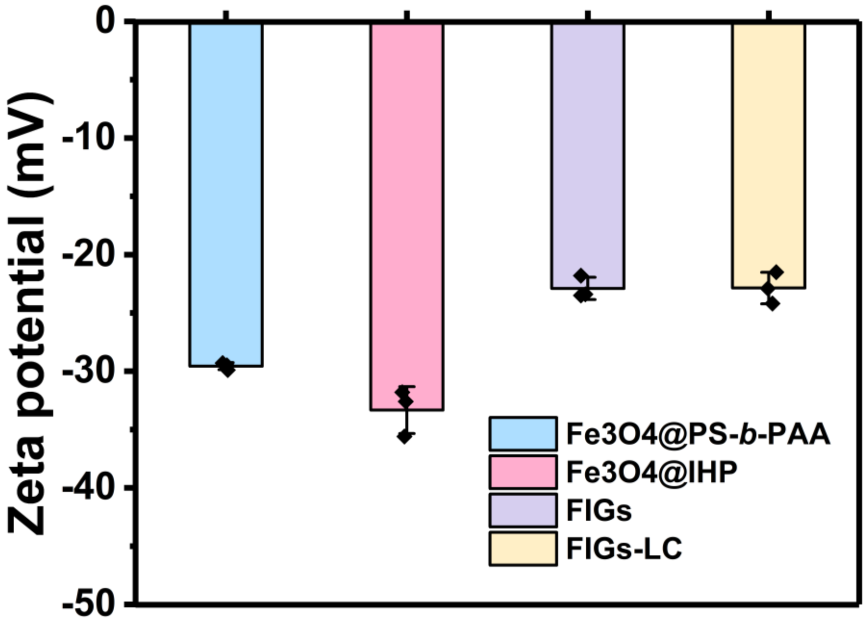

Supplementary Figure 5. Zeta potentials of Fe_3O_4@PS-*b*-PAA, Fe_3O_4@IHPs, FIGs, and FIGs-LC, respectively. Data are presented as mean values ± s.d. (n = 3).

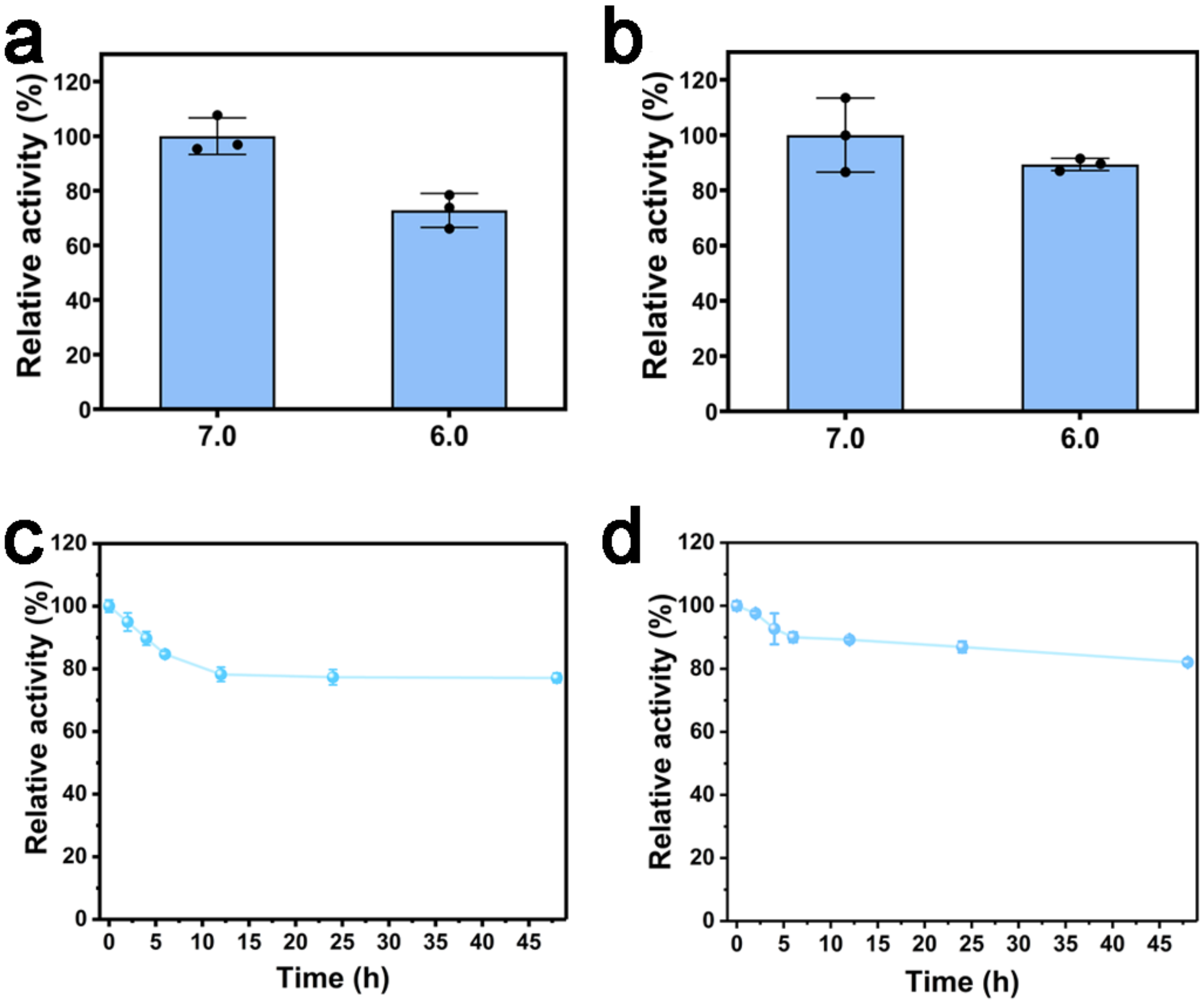

Supplementary Figure 6. a LOx activity of FIGs-L (without catalase) in the simulated TME (containing 100 μM of H_2O_2, 10 mM of GSH, and 2 mM of lactate) with different pH values. **b** CAT activity of FIGs-LC in the simulated TME with different pH values. **c** Storage activity of LOx in FIGs-LC at room temperature for 48 hours. **d** Storage activity of CAT in FIGs-LC at room temperature for 48 hours. Data are presented as mean values ± s.d. (*n* = 3).

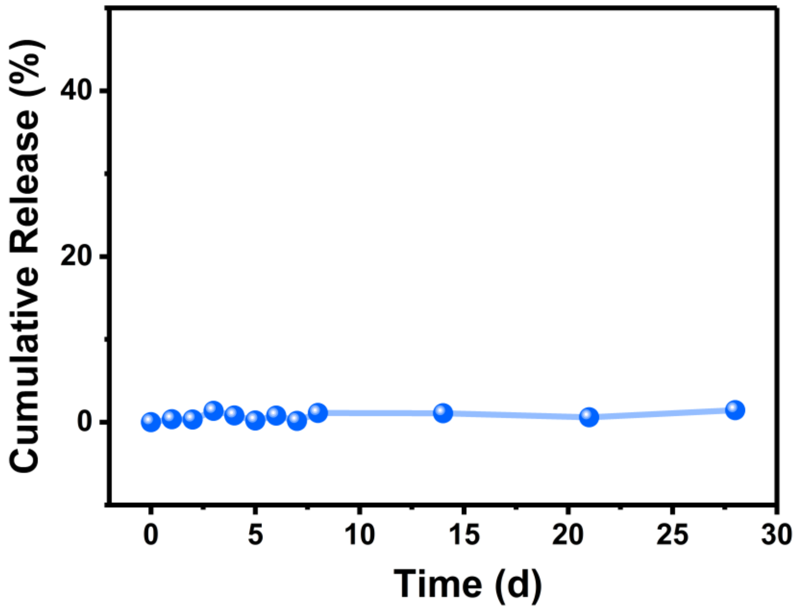

Supplementary Figure 7. The cumulative release of the enzymes in FIGs-LC in PBS at 37 °C.

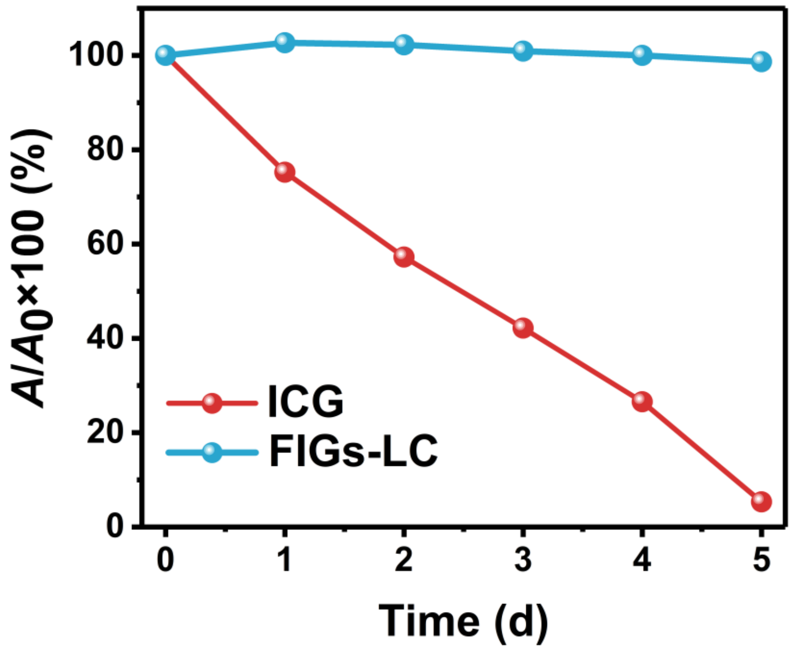

Supplementary Figure 8. Stability of free ICG and FIGs-LC in deionized water, reflected by the absorbance at 780 nm in UV-Vis-NIR absorption spectra.

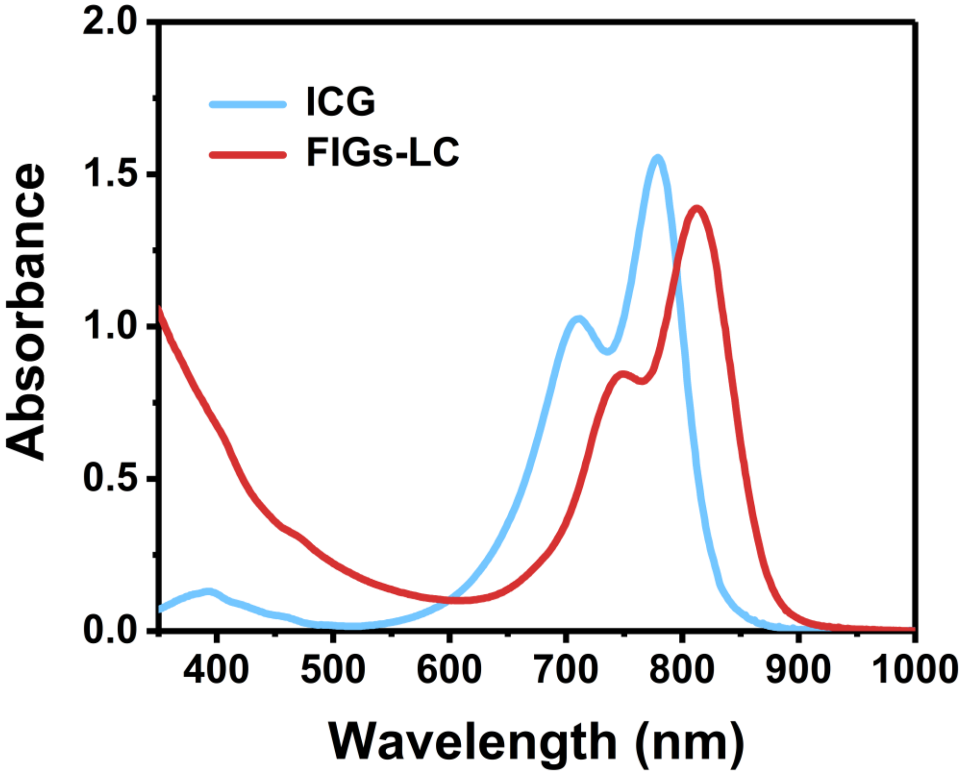

Supplementary Figure 9. UV-Vis-NIR absorption spectra of ICG and FIGs-LC.

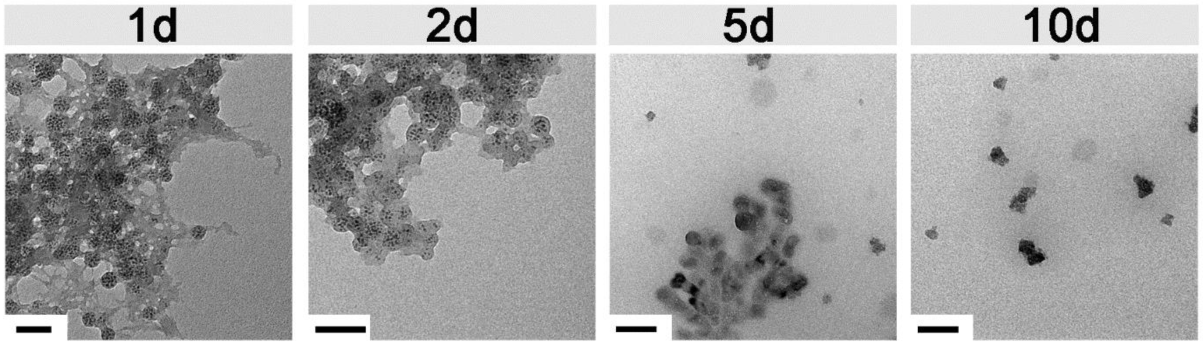

Supplementary Figure 10. TEM images of FIGs-LC after incubated in PBS (containing 10 mM GSH and 100 µM H_2O_2 and 2 mM lactate, pH 6.0) for 1 d, 2 d, 5 d, and 10 d. Scale bar:100 nm.

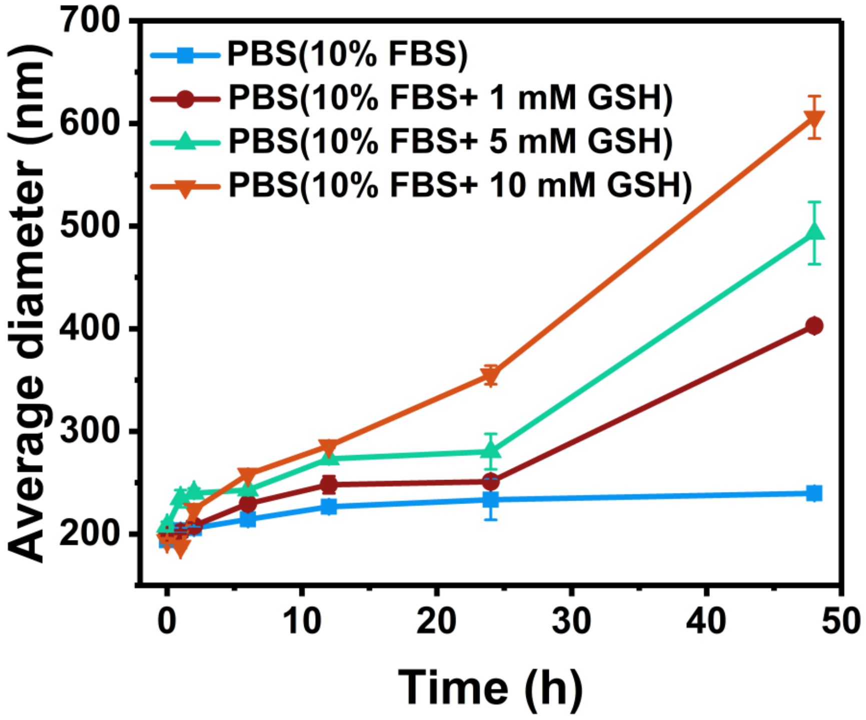

Supplementary Figure 11. Size changes (by Intensity) of FIGs-LC in PBS with different GSH concentrations. Data are presented as mean values ± s.d. (n = 3).

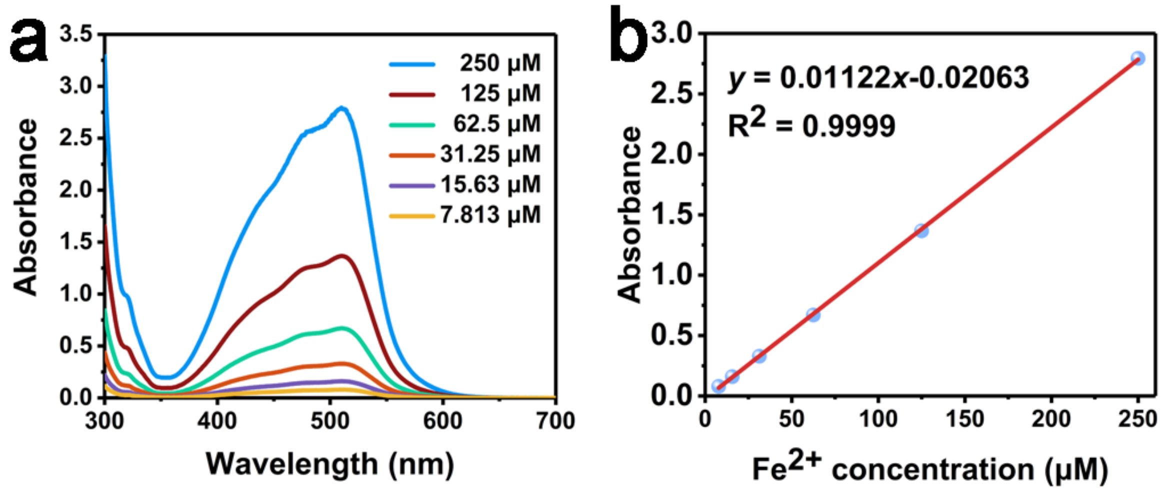

Supplementary Figure 12. The quantification of Fe^{2+} by using 1,10-phenanthroline monohydrate. (**a**) The UV-Vis spectra of Fe^{2+} solutions with different concentrations (7.813 μM, 15.63 μM, 31.25 μM, 62.50 μM, 125.00 μM, 250.00 μM) after adding 1,10-phenanthroline monohydrate. (**b**) The standard curve of Fe^{2+} for absorbance value at 510 nm. $y = 0.01122\ x$-0.02063, $R^2 = 0.9999$.

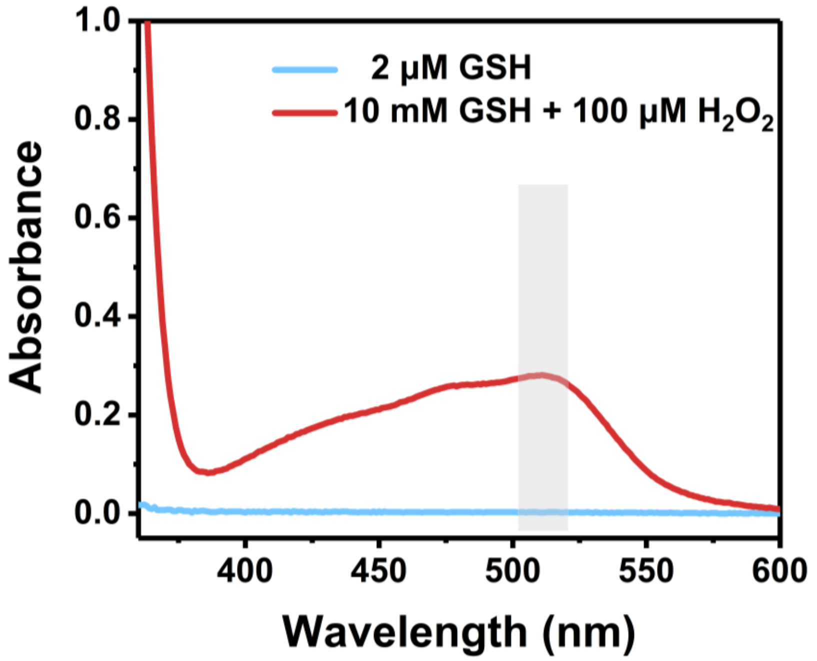

Supplementary Figure 13. The Fe^{2+} release under the simulated normal physiological conditions (2 μM GSH) and tumoral conditions (10 mM GSH and 100 μM H_2O_2), after incubation for 72 h, none of detectable Fe^{2+} was observed by the absorbance at 510 nm in the UV-Vis spectrum.

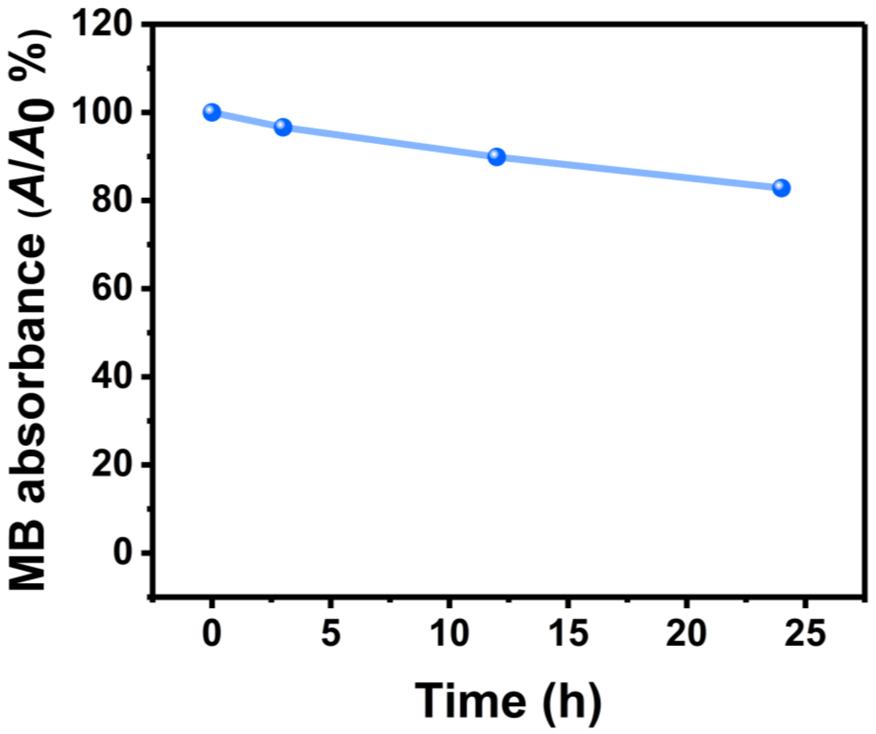

Supplementary Figure 14. The MB degradation curve under the simulated normal physiological conditions.

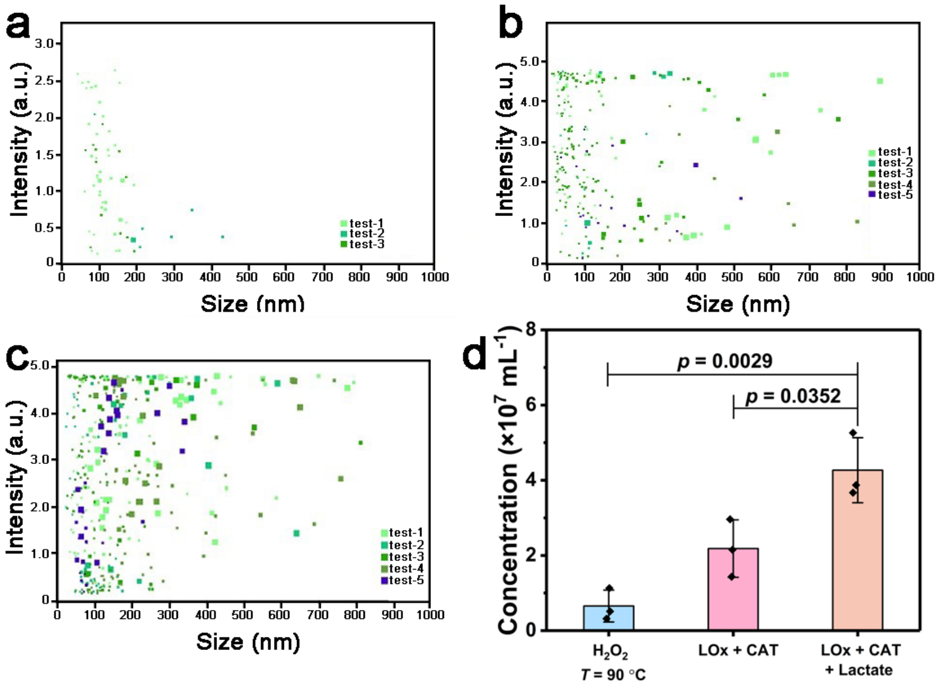

Supplementary Figure 15. Images of particle distribution under Brownian motion in different solutions, **(a)**: H_2O_2 (100 μM in PBS, pH=6, *T*=90 °C), **(b)**: LOx-CAT (PBS, pH=6), and **(c)**: LOx-CAT (PBS containing 100 μM lactate, pH=6). **(d)** The corresponding total nanoparticle concentrations. In **a**, **b**, **c**, the legends represent different replicates. Data represent mean ± s.d. from three independent replicates, *p* values were analyzed by Student's two-sided *t*-test.

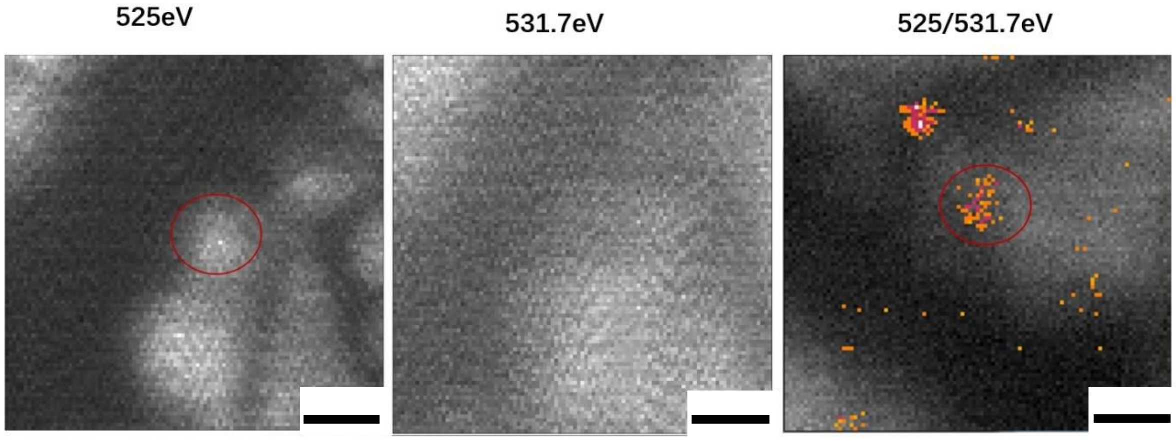

Supplementary Figure 16. High density of O_2 in the micro/nanobubbles: STXM transmission images of multiple micro/nanobubble at photon energies of 525 eV and 531.7 eV, as water barely absorbs soft X-rays below 535 eV (Shanghai Synchrotron Radiation Facility, Shanghai Institute of Applied Physics, Chinese Academy of Sciences). Scale bar: 2 μm

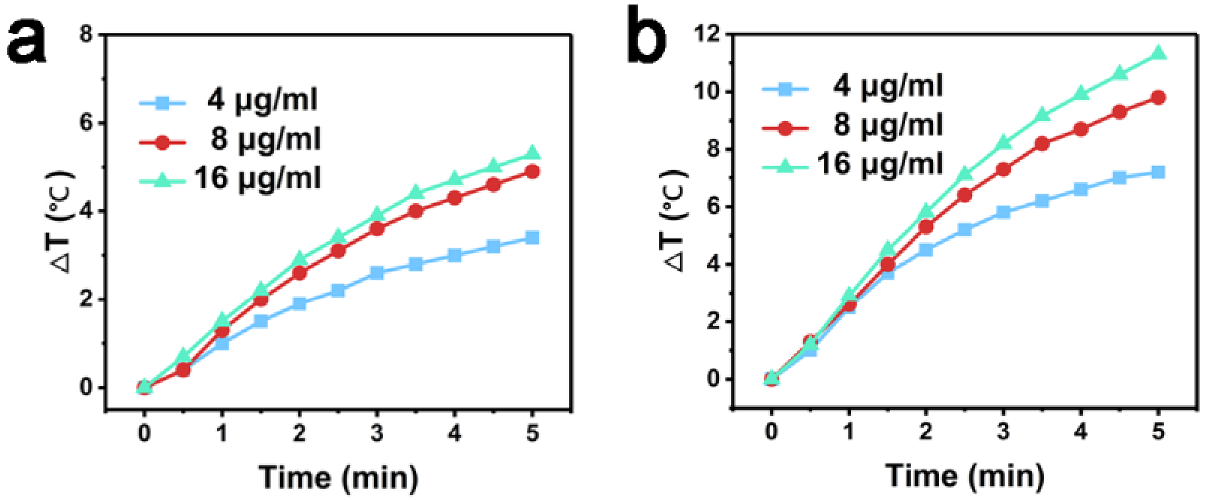

Supplementary Figure 17. Temperature elevation of FIGs-LC solutions with different ICG concentration under irradiation of an 808 nm light with the power of 0.25 W (**a**) and 0.5 W (**b**).

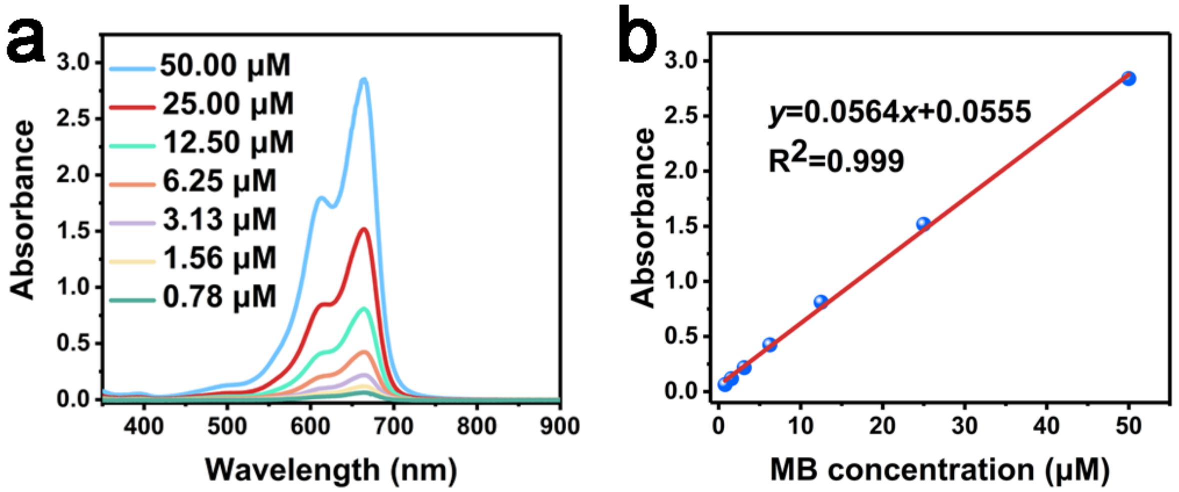

Supplementary Figure 18. (**a**) The UV-Vis spectra of MB solutions with different concentrations (0.78 μM, 1.56 μM, 3.13 μM, 6.25 μM, 12.50 μM, 25.00 μM, and 50.00 μM). (**b**) The standard concentration-absorbance curves of MB for absorbance value at 664 nm. $y = 0.0564\,x + 0.0555$, $R^2 = 0.999$.

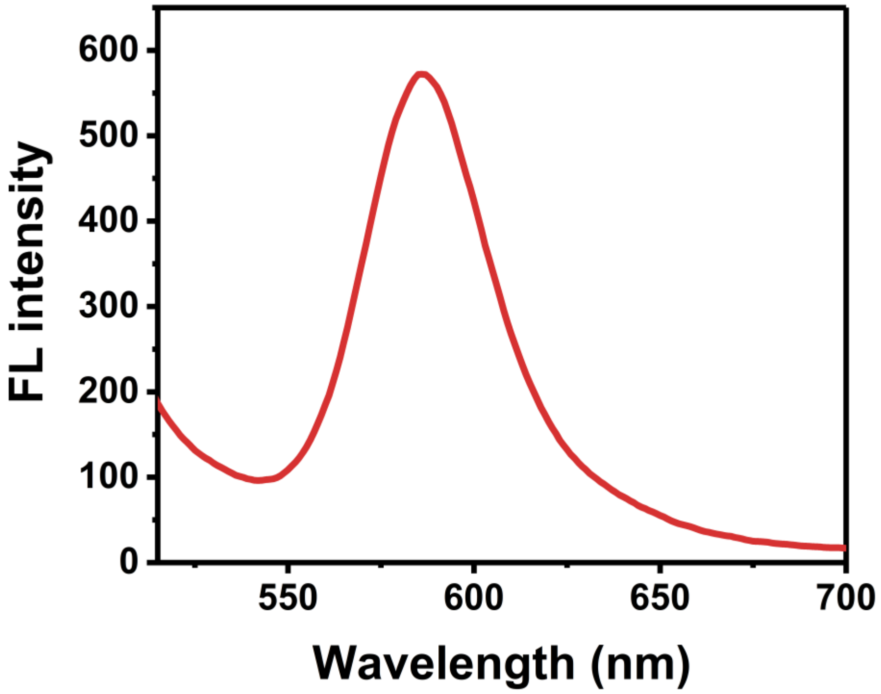

Supplementary Figure 19. Fluorescence emission spectrum of FRGs-LC, the characteristic peak at 585 nm is derived from Rhodamine B.

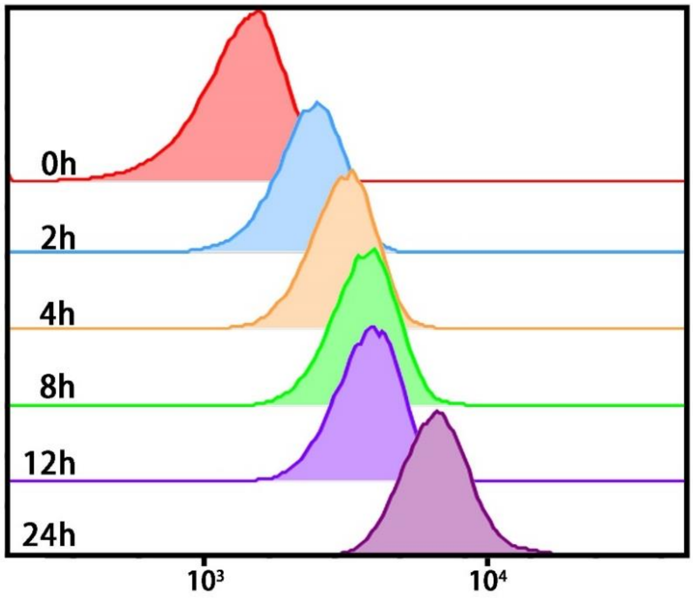

Supplementary Figure 20. Flow cytometry analysis of SMMC-7721 after incubation with FRGs-LC over different time.

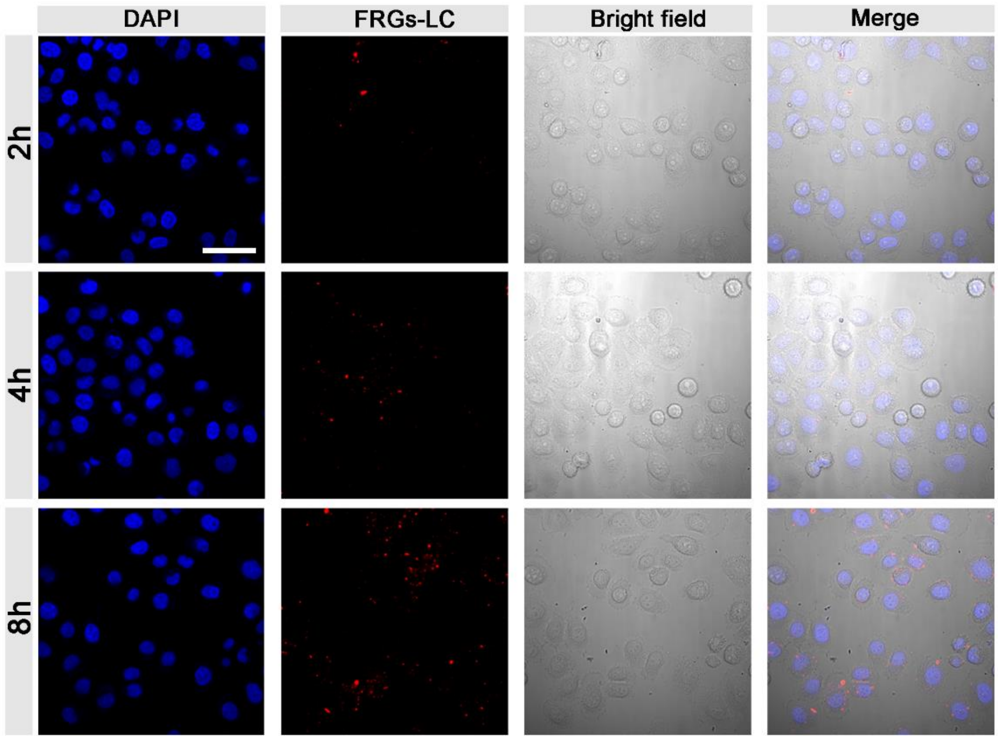

Supplementary Figure 21. CLSM images of intracellular Rhodamine B (RhB) for SMMC-7721 cancer cells incubated with FRGs-LC for 2 h, 4 h, and 8 h. Scale bar: 50 μm.

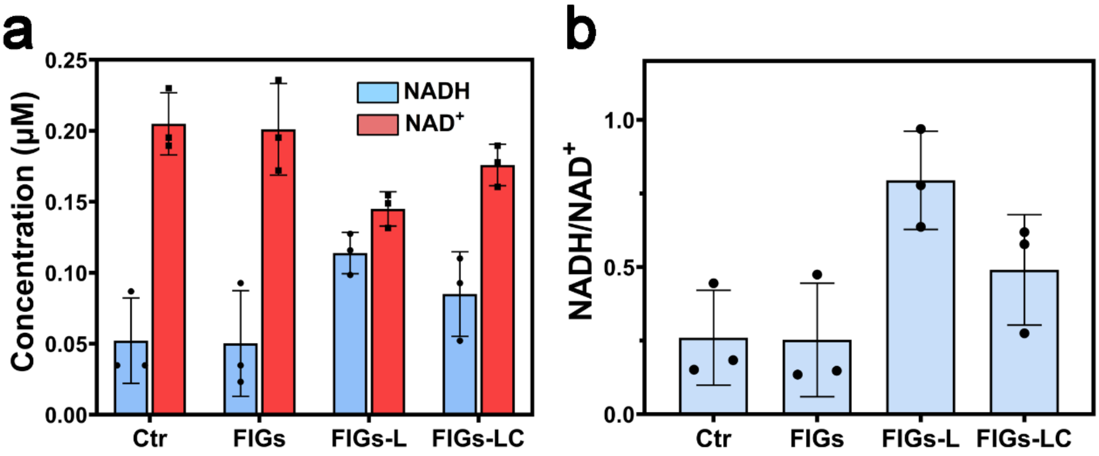

Supplementary Figure 22. Concentration of intracellular NADH and NAD^+ (**a**) and the NADH/NAD^+ redox ratio (**b**) of cells after co-incubation with FIGs, FIGs-L and FIGs-LC. Data represent mean ± s.d. from three independent replicates.

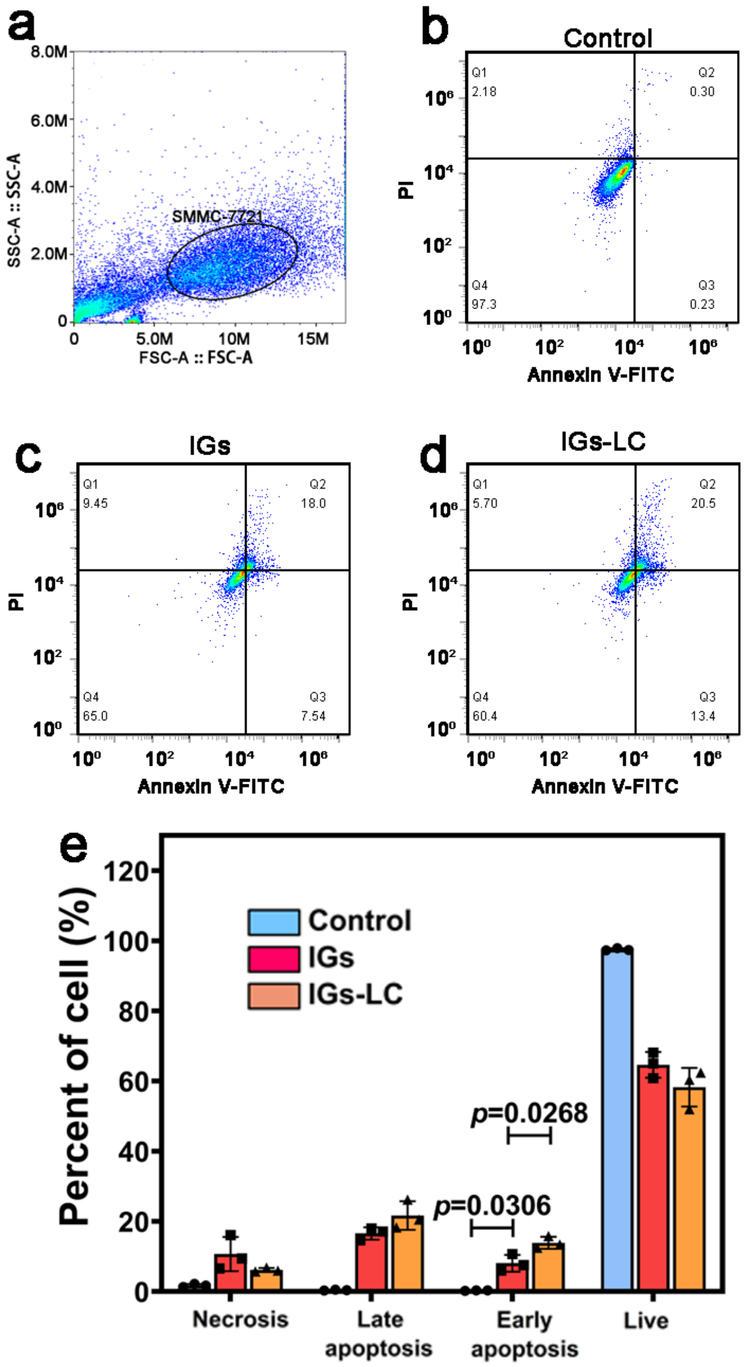

Supplementary Figure 23. a Gating strategy to analyze the apoptosis of cells. The apoptosis analysis of cells after co-incubation with: PBS (**b**, control), IGs (**c**) and IGs-LC (**d**). **e** Percent of cells in different stages (Necrosis, late apoptosis, early apoptosis, and live), data represent mean ± s.d. from three independent replicates, *p* values were analyzed by Student's two-sided *t*-test.

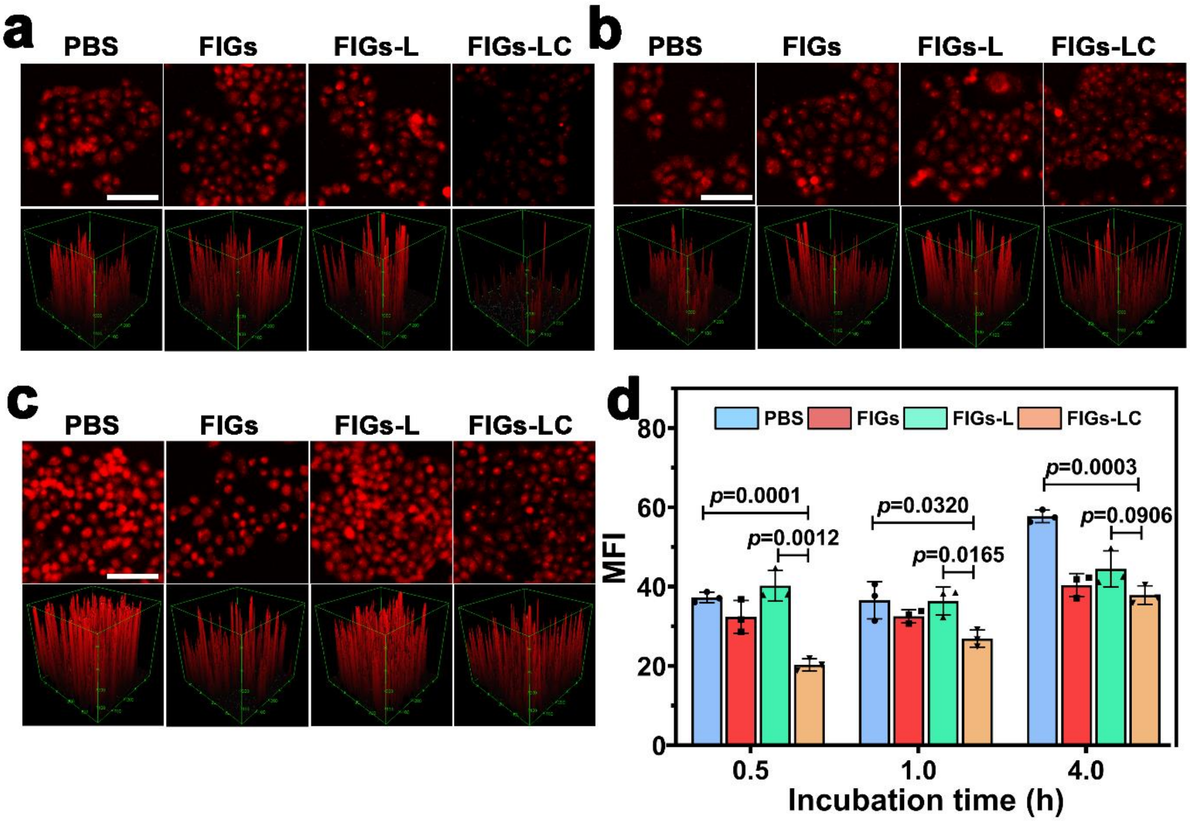

Supplementary Figure 24. Confocal images and corresponding surface plot images of the intracellular O_2 level indicator Ru(dpp)-stained SMMC-7721 cells after co-incubated with different solutions (PBS, FIGs, FIGs-L, FIGs-LC) for 0.5 h **(a)**, 1.0 h **(b)**, and 4.0 h **(c)** in hypoxic condition, scale bar: 100 μm. **d** The corresponding mean fluorescence intensity of Ru(dpp), data represent mean ± s.d. from three independent replicates, *p* values were analyzed by Student's two-sided *t*-test.

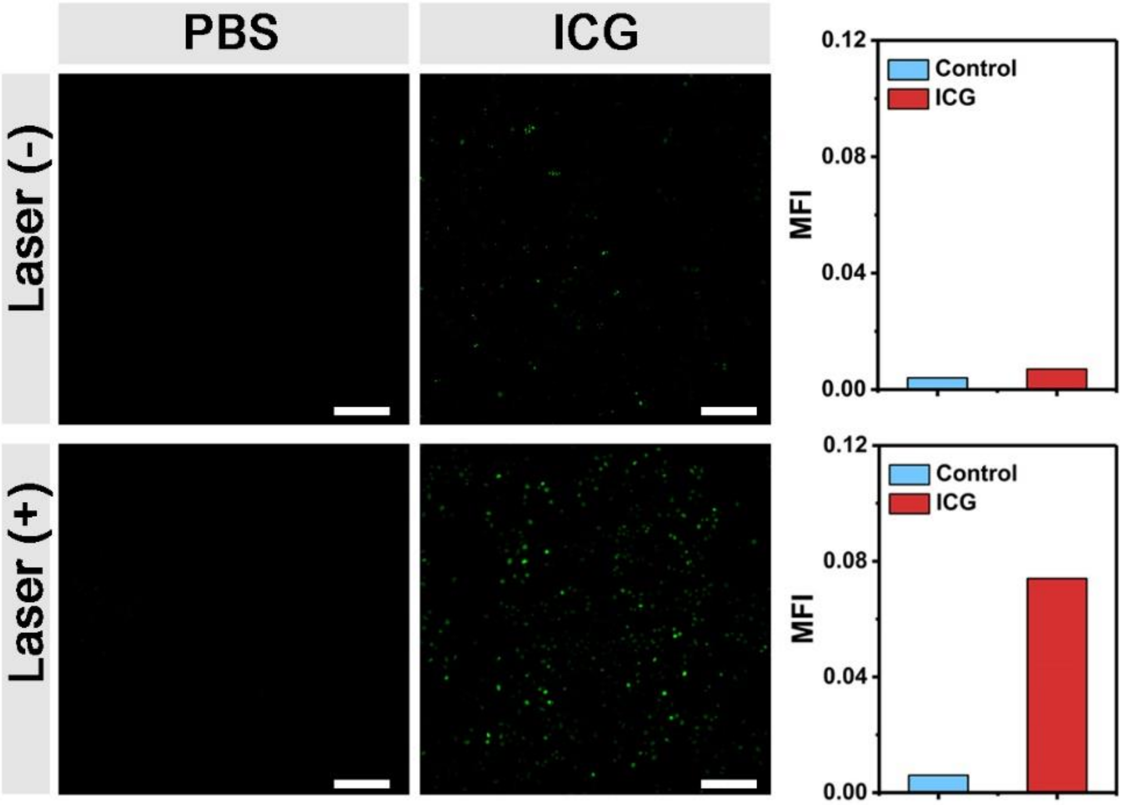

Supplementary Figure 25. CLSM images of SMMC-7721 cells treated with PBS and ICG for 12 h with DCFH-DA as a ROS detector, Scale bar: 200 μm. And the corresponding mean fluorescence intensity (MFI) of DCF fluorescence for ROS in cancer cells without laser irradiation, the data were analyzed by Image J.

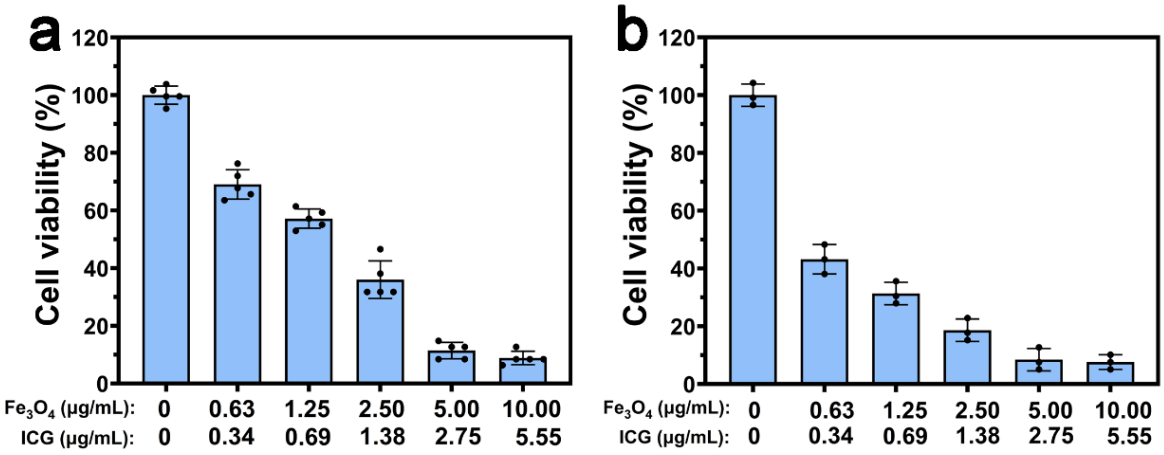

Supplementary Figure 26. The cytotoxicity of FIGs-L after the co-incubation with normal cells (3T3 cells) (**a**, lactate concentration: 0.5 mM, *n*=5) and tumor cells (SMMC-7721 cells) (**b**, lactate concentration: 2.0 mM, *n*=3). Data represent means ± s.d.

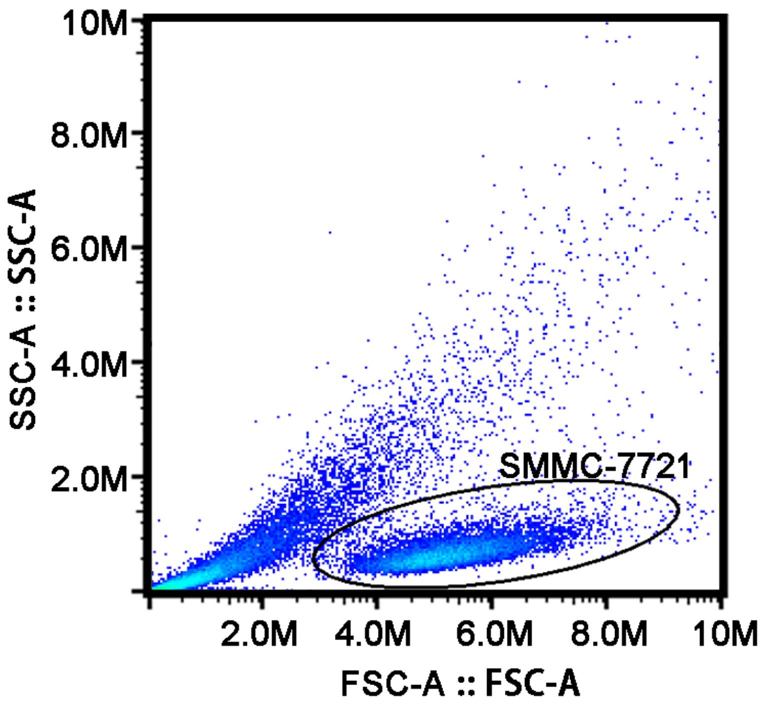

Supplementary Figure 27. Gating strategy to analyze the apoptosis of cells in Fig. 5h-k.

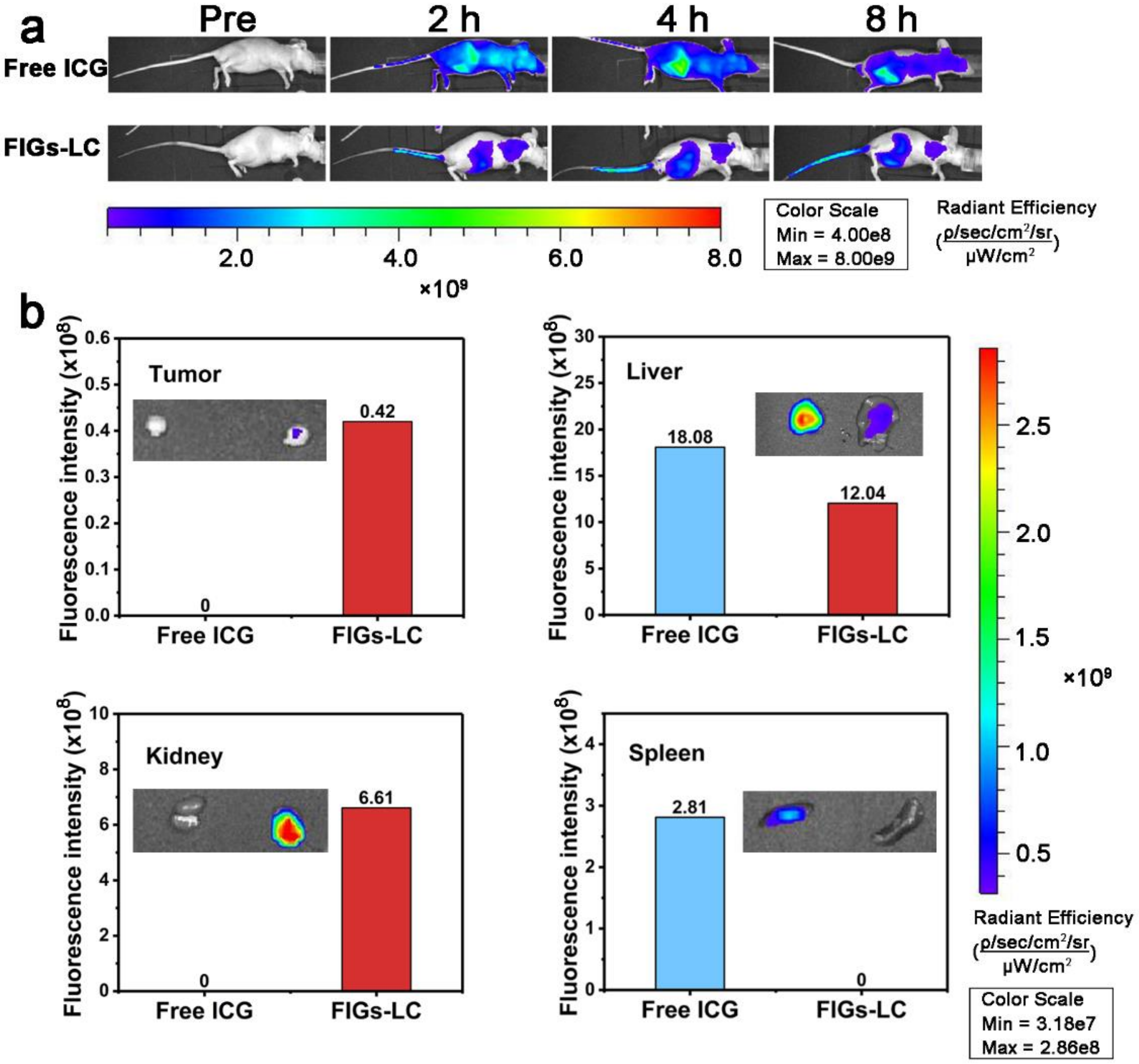

Supplementary Figure 28. (**a)** In vivo fluorescence images of tumor-bearing mice at 0 h, 2 h, 4 h, and 8 h after intravenous injection of ICG or FIGs-LC. (**b)** Ex vivo fluorescence images the semiquantitative analyses of tumors and organs of mice with intravenous injection of free ICG or FIGs-LC.

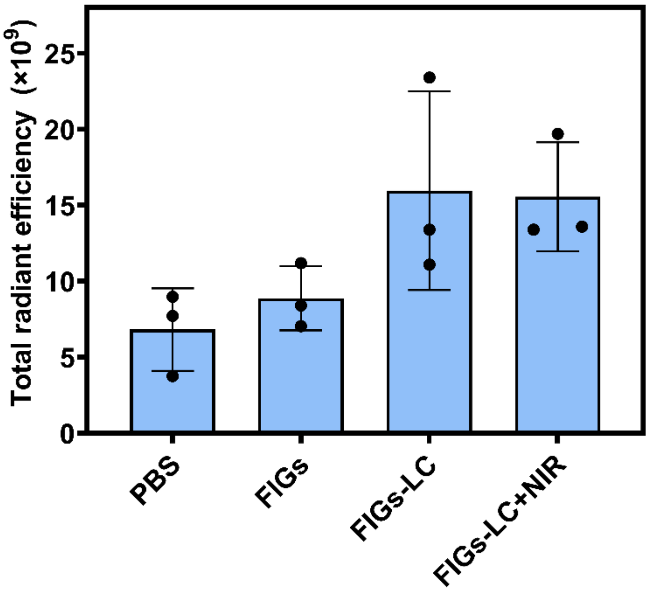

Supplementary Figure 29. The quantitative fluorescence data of ROS indicator DHE after co-injected with different solutions (PBS, FIGs, FIGs-LC) into tumor tissues and the NIR irradiation was conducted after 4 h, Ex=504 nm, Em=605 nm. Data represent mean ± s.d. from three independent replicates.

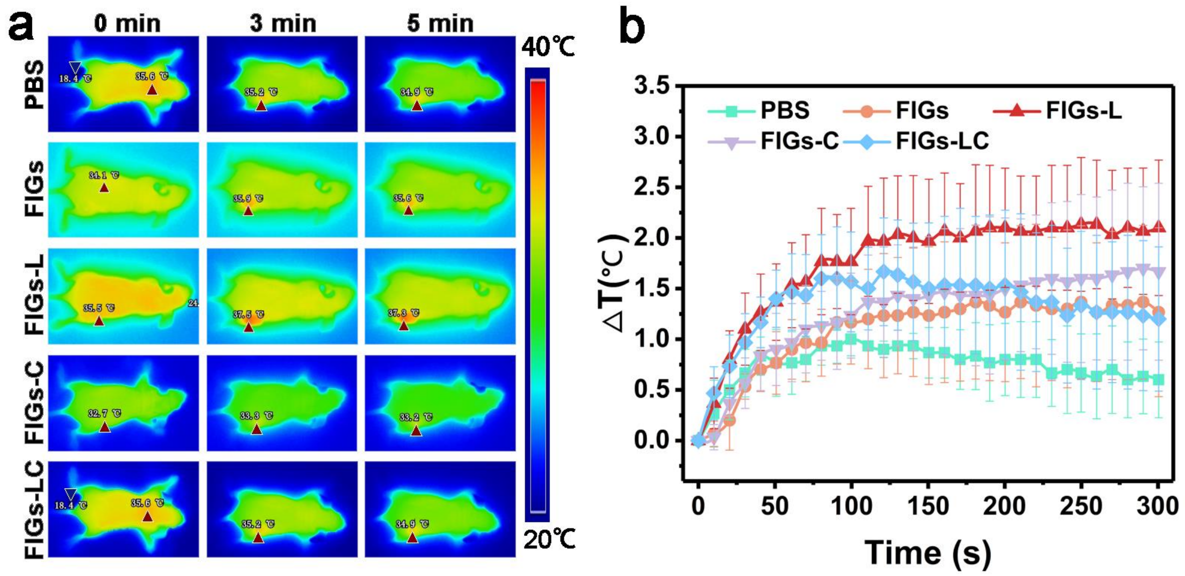

Supplementary Figure 30. (a) IR thermal images and **(b)** the corresponding heating curves of mice injected with PBS, FIGs, FIGs-L, FIGs-C, and FIGs-LC, irradiated with 808 nm laser (0.5 W), data represent mean ± s.d. from three independent replicates.

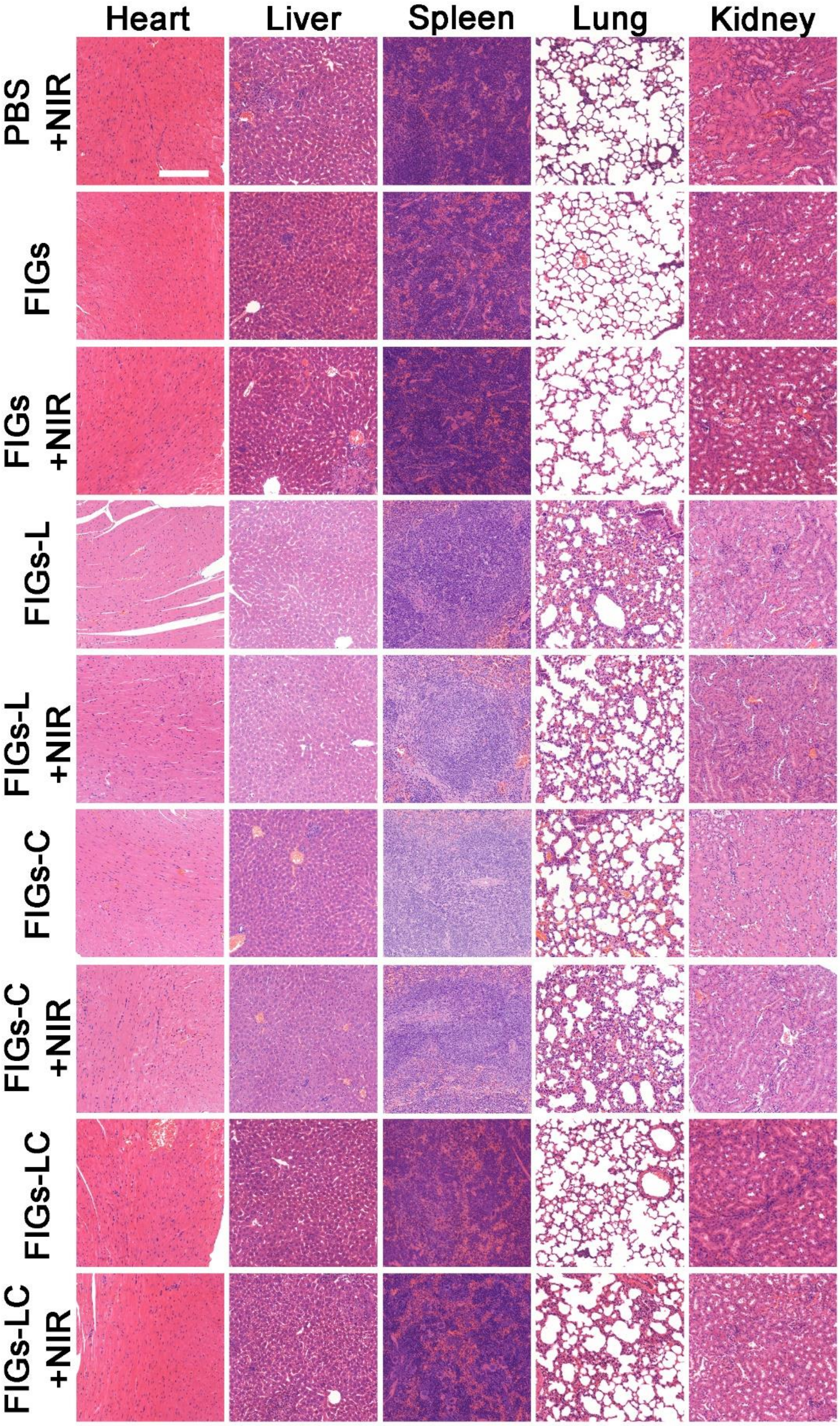

Supplementary Figure 31. H&E staining images of the major organs (heart, liver, spleen, lung, and kidney), scale bar: 200 μm.

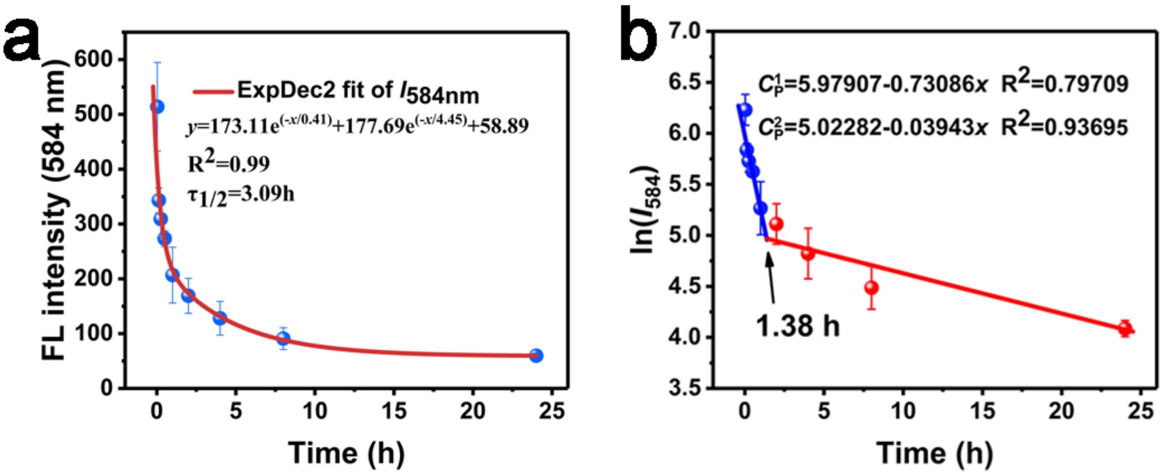

Supplementary Figure 32. (**a**) The blood circulation curve of intravenously injected FRGs-LC, based on the fluorescence intensity at 584 nm, the half-time ($\tau_{1/2}$) was calculated to be 3.09 h. (**b**) The eliminating rate curves of intravenously injected FRGs-LC from the blood circulation curve based on the ln (I_{584nm})-time relationship. The shifting interval of the two stages was calculated to be 1.38 h. Error bars = Standard Deviation (n=3).

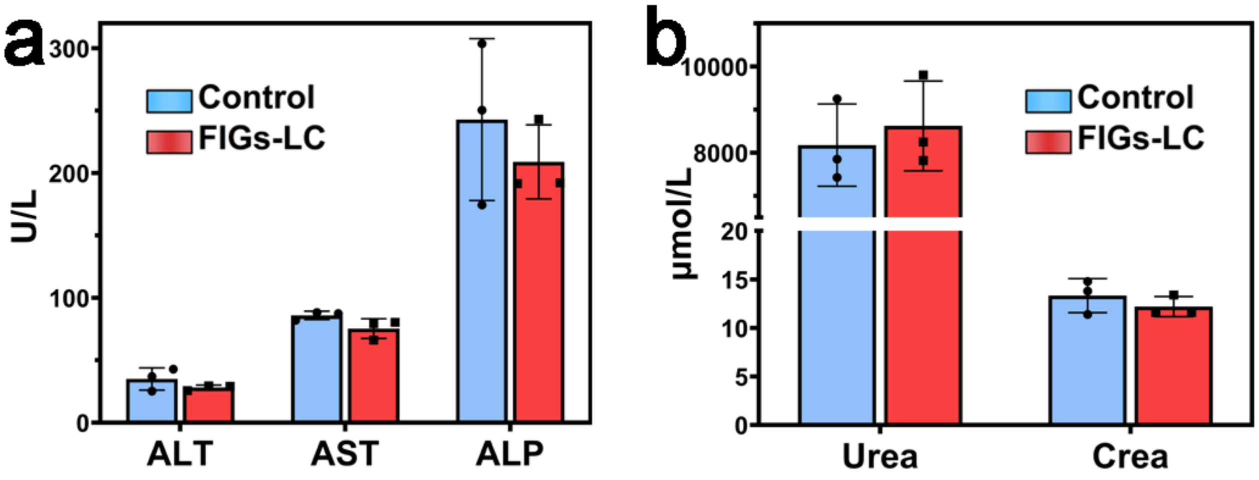

Supplementary Figure 33. (**a**) Blood biochemistry assays of liver function markers: alanine aminotransferase (ALT), aspartate aminotransferase (AST), alkaline phosphatase (ALP) in mice. (**b**) Blood biochemistry assays of kidney function markers: Urea and creatinine (Crea). Data represent mean ± s.d. from three independent replicates.

Supplementary Table 1. The loading efficiency and loading amount of LOx and CAT.

Enzyme	FIGs	Added enzyme	Residual (4 mL)	Loading	Loading efficiency	Loading amount
LOx	10 mg	20.5 U	4.96 U/mL	19.86 U	96.88 %	1.99 U/mg
CAT	10 mg	41.0 U	8.70 U/mL	34.78 U	84.83 %	3.48 U/mg

Supplementary Table 2. Hematology analysis of mice with FIGs-LC administration (*n*=3)

Test (units)	Control (mean ± SD)	FIGs-LC (mean ± SD)
WBC (10^9/L)	5.92 ± 2.29	9.90 ± 1.03
RBC (10^{12}/L)	8.83 ± 0.22	8.73 ± 0.22
HGB (g/L)	140.33 ± 3.68	143.67 ± 0.94
HCT (%)	41.37 ± 2.73	44.40 ± 0.80
MCV (fL)	46.80 ± 2.82	50.83 ± 0.50
MCH (pg)	15.90 ± 0.22	16.43 ± 0.31
MCHC (g/L)	340.33 ± 15.15	323.67 ± 3.68
PLT (10^9/L)	890.00 ± 421.91	1241.33 ± 188.24
RDW-SD (fL)	37.37 ± 0.62	36.17 ± 1.64
RDW-CV (%)	24.10 ± 1.23	21.77 ± 1.02
PDW (fL)	9.20 ± 0.46	8.43 ± 0.31
MPV (fL)	7.90 ± 0.30	7.67 ± 0.12
P-LCR (%)	12.10 ± 1.15	10.30 ± 0.86
PCT (%)	1.15 ± 0.22	0.95 ± 0.14
NEUT# (10^9/L)	2.46 ± 1.28	2.42 ± 0.87
LYMPH# (10^9/L)	3.30 ± 0.99	7.22 ± 0.17
MONO# (10^9/L)	0.01 ± 0.01	0.01 ± 0.01
EO# (10^9/L)	0.14 ± 0.07	0.24 ± 0.05
BASO# (10^7/L)	0.33 ± 0.47	0.33 ± 0.47
NEUT% (%)	39.93 ± 5.85	23.87 ± 5.83
LYMPH% (%)	57.57 ± 6.07	73.53 ± 5.80
MONO% (%)	0.23 ± 0.21	0.10 ± 0.08
EO% (%)	2.20 ± 0.37	2.47 ± 0.62
BASO% (%)	0.07 ± 0.09	0.03 ± 0.05
