# Supplementary material for: Peroxisome inspired hybrid enzyme nanogels for chemodynamic and photodynamic therapy
Source: Nat Commun. 2021 Sep 2;12:5243. doi: 10.1038/s41467-021-25561-z (PMC8413279; doi:10.1038/s41467-021-25561-z)
Supplement: Supplementary file 2 — Reporting Summary [file 41467_2021_25561_MOESM2_ESM.pdf]

nature portfolio

Corresponding author(s): Dechao Niu

Last updated by author(s): Jul 7, 2021

Reporting Summary

Nature Portfolio wishes to improve the reproducibility of the work that we publish. This form provides structure for consistency and transparency in reporting. For further information on Nature Portfolio policies, see our Editorial Policies and the Editorial Policy Checklist.

Statistics

For all statistical analyses, confirm that the following items are present in the figure legend, table legend, main text, or Methods section.

n/a	Confirmed	
☐	☒	The exact sample size (n) for each experimental group/condition, given as a discrete number and unit of measurement
☐	☒	A statement on whether measurements were taken from distinct samples or whether the same sample was measured repeatedly
☐	☒	The statistical test(s) used AND whether they are one- or two-sided<br>*Only common tests should be described solely by name; describe more complex techniques in the Methods section.*
☒	☐	A description of all covariates tested
☒	☐	A description of any assumptions or corrections, such as tests of normality and adjustment for multiple comparisons
☐	☒	A full description of the statistical parameters including central tendency (e.g. means) or other basic estimates (e.g. regression coefficient) AND variation (e.g. standard deviation) or associated estimates of uncertainty (e.g. confidence intervals)
☐	☒	For null hypothesis testing, the test statistic (e.g. F, t, r) with confidence intervals, effect sizes, degrees of freedom and P value noted<br>*Give P values as exact values whenever suitable.*
☒	☐	For Bayesian analysis, information on the choice of priors and Markov chain Monte Carlo settings
☒	☐	For hierarchical and complex designs, identification of the appropriate level for tests and full reporting of outcomes
☒	☐	Estimates of effect sizes (e.g. Cohen's d, Pearson's r), indicating how they were calculated

Our web collection on statistics for biologists contains articles on many of the points above.

Software and code

Policy information about availability of computer code

Data collection	The hydrodynamic sizes and zeta potentials of nanoparticles were measured with a Malvern Zetasizer Nano Series. Fourier transform infrared (FT-IR) spectra were recorded with a Thermo Scientific Nicolet 6700. Raman shift spectra were recorded with a Renishaw Invia Reflex Laser Micro-Raman Spectrometer with 785 nm laser. Transmission electron microscopy (TEM) images were taken by a JEM 2100F electron microscope operated at 200 kV and JEM 1400 electron microscope operated at 100 kV. Emission spectra were recorded with a Shimadzu Fluorescence Spectrophotometer RF-5301PC (1 cm quartz cell). Flow cytometry (FC) was conducted by BD Accuri C6. Confocal laser scanning microscope (CLSM) images were taken by Nikon A1 Confocal Laser Scanning Microscopy (CLSM). UV-Vis-NIR spectra were measured with a Shimadzu spectrophotometer UV-3600 (1 cm quartz cell). Thermogravimetric analyses (TG) were conducted on a thermal analyzer (PerkinElmer TGA 8000). Electron spin resonance was measured with a Bruker EMX-8/2.7 (100G-18 KG) spectrometer. Nanobubbles were tested by the NanoSight technology analysis (NTA, Claire Hannell, NanoSight Limited, Salisbury, UK).
Data analysis	Microsoft Excel 2019, GraphPad prism 8, and Origin 2017 were used for statistical analysis. Flowjo_V10 was used for flow cytometry. Image J 1.52p were used for the semiquantitativeanalyses of CLSM images.

For manuscripts utilizing custom algorithms or software that are central to the research but not yet described in published literature, software must be made available to editors and reviewers. We strongly encourage code deposition in a community repository (e.g. GitHub). See the Nature Portfolio guidelines for submitting code & software for further information.

Data

Policy information about availability of data

All manuscripts must include a data availability statement. This statement should provide the following information, where applicable:

- Accession codes, unique identifiers, or web links for publicly available datasets
- A description of any restrictions on data availability
- For clinical datasets or third party data, please ensure that the statement adheres to our policy

The experimental data supporting the findings of this study are available within the article, Supplementary Information, and Source Data. Additional data are available from the corresponding authors upon reasonable request. Source data are provided with this paper.

Field-specific reporting

Please select the one below that is the best fit for your research. If you are not sure, read the appropriate sections before making your selection.

☒ Life sciences ☐ Behavioural & social sciences ☐ Ecological, evolutionary & environmental sciences

For a reference copy of the document with all sections, see nature.com/documents/nr-reporting-summary-flat.pdf

Life sciences study design

All studies must disclose on these points even when the disclosure is negative.

Sample size	Sample size of n ≥ 3 for cell and animal experiments were chosen for statistic analysis.
Data exclusions	No data were excluded from the analyses.
Replication	Sample were synthesized independently for characterization and analysis.
Randomization	The cells and animal used in this paper were randomly distributed into different groups for experiments when the cells were in logarithmic growth or tumor volumes reached the proper size (about 100 mm3).
Blinding	The investigators were blinded to group allocation during experiments, data collection and analysis.

Reporting for specific materials, systems and methods

We require information from authors about some types of materials, experimental systems and methods used in many studies. Here, indicate whether each material, system or method listed is relevant to your study. If you are not sure if a list item applies to your research, read the appropriate section before selecting a response.

Materials & experimental systems

n/a	Involved in the study	
☒	☐	Antibodies
☐	☒	Eukaryotic cell lines
☒	☐	Palaeontology and archaeology
☐	☒	Animals and other organisms
☒	☐	Human research participants
☒	☐	Clinical data
☒	☐	Dual use research of concern

Methods

n/a	Involved in the study	
☒	☐	ChIP-seq
☐	☒	Flow cytometry
☒	☐	MRI-based neuroimaging

Eukaryotic cell lines

Policy information about cell lines

Cell line source(s)	Mouse embryo fibroblast (NIH-3T3) cells and human hepatocellular carcinoma SMMC-7721 cells were purchased from Jiangsu Keygen Biotech Corp., Ltd.
Authentication	These cells were authenticated by cell vitality test, isozyme detection, and mycoplasma detection.
Mycoplasma contamination	The cell lines were detected for mycoplasma contaminaiton and no mycoplasma was found.
Commonly misidentified lines (See ICLAC register)	None of these cell lines were used

Animals and other organisms

Policy information about studies involving animals; ARRIVE guidelines recommended for reporting animal research

Laboratory animals	Female Balb/c nude mice (6 weeks old) and Kunming mice (5 weeks old) were used in this paper. All mice were housed in a specific pathogen-free environment at 26 ± 1 °C and 50 ± 5% humidity, with a 12 h light-dark cycle.
Wild animals	No wild animals
Field-collected samples	No field-collected samples.
Ethics oversight	All animals experiments were conducted with the approval of Animal Ethics Committee of East China University of Science and Technology, and under the Guidance for Care and Use of Laboratory Animals of East University of Science and Technology, animal experiments were carried out.

Note that full information on the approval of the study protocol must also be provided in the manuscript.

Flow Cytometry

Plots

Confirm that:

- [x] The axis labels state the marker and fluorochrome used (e.g. CD4-FITC).
- [x] The axis scales are clearly visible. Include numbers along axes only for bottom left plot of group (a 'group' is an analysis of identical markers).
- [x] All plots are contour plots with outliers or pseudocolor plots.
- [x] A numerical value for number of cells or percentage (with statistics) is provided.

Methodology

Sample preparation	SMMC-7721 cells were seeded in 6-well plates at a density of 1.0x105 cells/well, after 24 hours, the cells were incubated with FIGs or FIGs-LC (at the Fe3O4 concentration of 10 "g/mL) for 12 h, then irradiated with an 808 nm laser (0.25 W) for 5 min, and incubated for another 36 h. Following the incubation with 5 "l of annexin V-FITC and PI at room temperature for 15 min. Finally, the cells were washed and trypsinized for flow cytometry analysis.
Instrument	BD Acurri C6 cytometer
Software	BD Accuri C6 Software and Flowjo_V10 were used for flow cytometry analysis.
Cell population abundance	The absolute cells around 10000 were analyzed for each group.
Gating strategy	Initial cell populations were gated for a live population using FSC and SSC plot of cell only sample. The gate was set to remove cell debris and dead cells.

- [x] Tick this box to confirm that a figure exemplifying the gating strategy is provided in the Supplementary Information.
